# A High‐Density Hydrogen Bond Locking Strategy for Constructing Anisotropic High‐Strength Hydrogel‐Based Meniscus Substitute

**DOI:** 10.1002/advs.202310035

**Published:** 2024-03-21

**Authors:** Qian Zhang, Xuxuan Yang, Kuan Wang, Ziyang Xu, Wenguang Liu

**Affiliations:** ^1^ School of Materials Science and Engineering, Tianjin Key Laboratory of Composite and Functional Materials Tianjin University Tianjin 300350 China

**Keywords:** 3D printing, anisotropic hydrogel, hydrogen bonds, meniscus substitute, preloading

## Abstract

Mimicking anisotropic features is crucial for developing artificial load‐bearing soft tissues such as menisci). Here, a high‐density hydrogen bond locking (HDHBL) strategy, involving preloading a poly(N‐acryloylsemicarbazide) (PNASC) hydrogel with an aqueous solution containing a hydrogen bond breaking agent, followed by water exchange, to fabricate anisotropic high‐strength hydrogels are proposed. During this process, multiple high‐density hydrogen bonds of the PNASC network are re‐established, firmly freezing oriented molecular chains, and creating a network with an anisotropic microstructure. The resulting anisotropic hydrogels exhibit superior mechanical properties: tensile strength over 9 MPa, Young's modulus exceeding 120 MPa along the orientation direction, and fatigue thresholds exceeding 1900 J m^−2^. These properties meet the mechanical demands for load‐bearing tissue substitutes compared to other reported anti‐fatigue hydrogels. This strategy enables the construction of an anisotropic meniscal scaffold composed of circumferentially oriented microfibers by preloading a digital light processing‐3D printed PNASC hydrogel‐based wedge‐shaped construct with a resilient poly(N‐acryloyl glycinamide) hydrogel. The 12‐week implantation of a meniscus scaffold in rabbit knee joints after meniscectomy demonstrates a chondroprotective effect on the femoral condyle and tibial plateau, substantially ameliorating the progression of osteoarthritis. The HDHBL strategy enables the fabrication of various anisotropic polymer hydrogels, broadening their scope of application.

## Graphical abstract

A high‐density hydrogen‐bond locking strategy is proposed for customizing circumferentially anisotropic meniscal skeletons. It involves preloading digital light processing 3D‐printed highly stiff gels, followed by water exchange to reconstruct multiple hydrogen bonds, freezing the molecular chains and anisotropic microstructures. Biomimetic meniscus scaffolds, fabricated by infilling the resilient hydrogel into a stiff skeleton, efficiently protect articular cartilage and alleviate osteoarthritis progression.

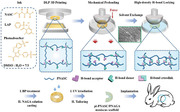

## 1 Introduction

The meniscus is a wedge‐shaped fibrocartilaginous tissue that plays an indispensable role in transferring and redistributing loads within the knee joint. It enhances congruency between the femoral condyle and tibial plateau, thereby maintaining the joint stability.^[^ ^1^, ^2^ ^]^ The meniscus tissue is highly susceptible to injury and has limited natural healing capacity. Clinically, a total meniscectomy is performed to relieve pains when irreparable meniscal tears occur.^[^ ^3^, ^4^ ^]^ However, this procedure leads to an undesirable and abrupt increase in intra‐articular contact stress, which can cause cartilage degeneration and subsequent joint issues such as osteoarthritis.^[^ ^5^, ^6^, ^7^, ^8^ ^]^ Therefore, the development of a meniscal substitute is imperative to restore knee biomechanics and alleviate articular cartilage wear.

Although a variety of meniscal substitutes have been developed and commercialized, most of them feature homogeneous structures and lack adequate biomechanical properties,^[^ ^9^, ^10^, ^11^ ^]^ limiting their effectiveness in achieving long‐term functional restoration. Anatomically, the meniscus is primarily composed of a circumferentially and radially aligned collagen fiber framework interlaced with hydrophilic glycosaminoglycans (GAGs).^[^ ^12^, ^13^ ^]^ Specifically, anisotropic collagen fibers provide mechanical support, particularly in providing circumferential tensile resistance, while GAGs play essential roles in energy absorption and water retention. Together, these components contribute to the biomechanical function of the menisci. In our previous study, a 3D printed high‐strength poly(N‐acryloyl glycinamide) (PNAGA) supramolecular polymer hydrogel‐cushioned radially and circumferentially oriented poly(ɛ‐caprolactone) (PCL) meniscal substitute (PCL‐PNAGA) was designed and constructed to replicate the microstructure and functions of the native meniscus. In vivo implantation experiments conducted over 12 weeks in a rabbit model demonstrated its effective chondroprotective function.^[^ ^14^ ^]^ However, the comprehensive mechanical properties of the PCL‐PNAGA meniscal substitute are still inferior to those of the natural meniscus, and its anisotropic features are limited to the macroscale. To date, the customization of a meniscus substitute mimicking the complex microstructure remains a significant challenge because of the difficulty in recapitulating circumferential anisotropy.

Anisotropic microstructures are prevalent in load‐bearing soft tissues such as menisci, ligaments, and tendons, contributing significantly to their biomechanical function within the human body. Recent research has demonstrated that the incorporation of anisotropic structures into scaffolds can enhance their resistance to fatigue fractures under repeated biomechanical loads. Therefore, fabricating tissue substitutes with anisotropic microstructures is desirable. Several methodologies have been proposed to achieve this goal. Mechanical training,^[^ ^15^, ^16^ ^]^ drying in confined conditions,^[^ ^17^, ^18^ ^]^ prestretching,^[^ ^19^, ^20^ ^]^ electric/magnetic field‐assisted assembly,^[^ ^21^, ^22^ ^]^ and directional freezing,^[^ ^23^, ^24^ ^]^ have been proposed for constructing anisotropic hydrogels. However, the majority of these reported methods mainly aim to replicate the uniaxial orientation characteristics of tendons, overlooking the more complex circumferential anisotropy observed in the meniscus. To the best of our knowledge, there is a paucity of studies on exploring methods for inducing circumferential alignment of molecular chains. Additionally, regardless of the chosen approach to impose molecular chain orientation, a stable and robust freeze mechanism is essential for preserving the anisotropic microstructure within the hydrogel network. In particular, unlike natural polymers with rigid or semi‐rigid molecular chains (e.g., cellulose), which easily respond to external forces for orientation and form stable anisotropic microfiber structures, the oriented conformation of flexible polymer chains tends to revert to random coils once the applied force is removed due to entropic elasticity. This challenge can be addressed by employing an effective freeze mechanism. Ion crosslinking and crystallization are commonly employed methods to freeze molecular chains and maintain the anisotropic microstructure of flexible polymer chains. Nonetheless, ion crosslinking can readily dissociate in physiological salt environments, and hydrogels stabilized with crystalline structures are limited to a few polymers with regular structures, e.g., polyvinyl alcohol (PVA). To expand the application scope of hydrogels in the construction of load‐bearing soft tissue substitutes, it is crucial to establish a universal and robust mechanism for freezing the oriented molecular chains.

**Scheme 1:**
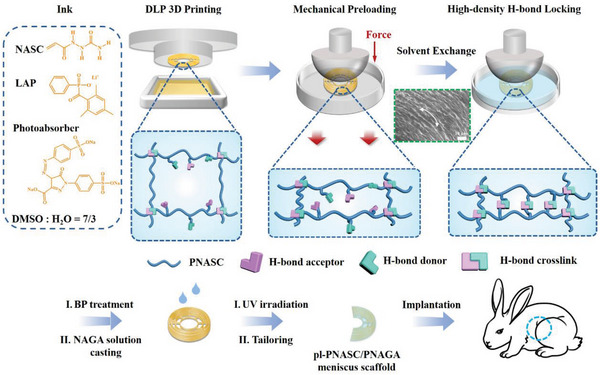
Schematic illustration of the high‐density hydrogen bond locking strategy for constructing 3D printed circumferentially anisotropic meniscus replacement by mechanical preloading and solvent exchange‐induced hydrogen bond reconstruction.

Nature harbors a vast diversity of remarkably distinct secondary structures, such as the double helix of deoxyribonucleic acid (DNA) and the β‐sheet of proteins, which are maintained in a stable manner due to the presence of multiple hydrogen bonds between base pairs or peptide bonds. Inspired by this, we developed a series of high‐strength hydrogels crosslinked by multiple hydrogen bonds. Recently, we prepared a supramolecular polymer hydrogel using poly(N‐acryloylsemicarbazide) (PNASC) through free radical polymerization of the N‐acryloylsemicarbazide (NASC) monomer in a solvent mixture of dimethyl sulfoxide (DMSO) and water, followed by solvent exchange in deionized water. Indeed, we initially discovered that significant hydrophobic phase separation occurred and no hydrogel formed after the polymerization of NASC in water due to the strong hydrogen bonding interaction of PNASC. Therefore, DMSO serving as hydrogen bonding dissociation agent was introduced into the system to weaken the strength of hydrogen bonds between PNASC. However, after the polymerization of NASC in pure DMSO solvent system, rapid reconstruction of high‐density hydrogen bonds caused by subsequent solvent exchange from DMSO to water also resulted in obvious phase separation, thus leading to inferior mechanical properties of PNASC hydrogel. In contrast, a small proportion of water was introduced into the mixed solvent for preparing the initial PNASC gel, which restricted the rapid aggregation of PNASC chains during the solvent exchange process due to already existing partial hydrogen bonds, thus inhibiting the occurrence of phase separation. Upon replacement of DMSO molecules with water molecules, the high‐density intermolecular hydrogen bonds among the PNASC amide/urea groups in the side chains were reconstructed, resulting in exceptional mechanical strength and resistance to swelling exhibited by the PNASC hydrogel. In particular, its Young's modulus (up to 100 MPa) was comparable to that of the native meniscus (circumferential tensile modulus of ≈ 113.0 MPa).^[^ ^25^, ^26^, ^27^ ^]^ The mechanism of solvent exchange‐induced multiple hydrogen bond reconstruction described above prompted us to explore the possibility of employing high‐density multiple hydrogen bonds as “locks” to freeze the conformation of oriented molecular chains, enabling the customization of anisotropic hydrogels through hydrogen bond crosslinking. Based on this hypothesis, we propose a high‐density hydrogen bond locking (HDHBL) strategy for designing anisotropic PNASC hydrogels with remarkable properties by combining mechanical preloading (a common method to force molecular chain orientation) with solvent exchange‐induced multiple hydrogen bond reconstruction (**Scheme** **1**). First, the PNASC gel impregnated with DMSO/water (referred to PNASC DMSO/water gel) was subjected to preloading (such as prestretching), followed by deformation fixation for 24 h. During this process, due to the dynamic nature of hydrogen bonds, the polymer chains were forced to orient along a predesigned direction and undergo further aggregation.^[^ ^28^ ^]^ Subsequently, the preloaded PNASC DMSO/water gel was immersed in the deionized water for solvent exchange, inducing the reconstruction of the multiple hydrogen bonds to “lock” the oriented polymer chains in place. This process results in the formation of anisotropic PNASC hydrogels with excellent mechanics. In this work, to validate the effectiveness of the HDHBL strategy, we initially utilized the simplest preloading mode (i.e., prestretching) to construct anisotropic PNASC hydrogels and characterized their structural features and mechanical performance. Subsequently, we investigated the versatility of the proposed strategy for constructing different anisotropic hydrogels. More importantly, we integrated the HDHBL strategy with digital light processing (DLP)−3D printing technology, enabling the circumferential alignment of molecular chains to emulate a meniscus for the first time. This involved applying a more intricate preloading technique using a customized platen on a meticulously designed DLP 3D‐printed wedge‐shaped PNASC DMSO/water gel‐based meniscus frame with both circumferential and radial beams. Subsequently, solvent exchange was applied to further customize the meniscal skeleton (pl‐PNASC) with a circumferential anisotropic microstructure. Furthermore, we incorporated the high strength non‐swelling PNAGA hydrogel into the resulting anisotropic meniscal skeleton to fabricate a pl‐PNASC/PNAGA hydrogel‐based meniscus scaffold mimicking the anisotropic mechanical properties of the natural meniscus. Finally, the resulting pl‐PNASC/PNAGA meniscus scaffold after swelling equilibrium was implanted into rabbit knee joints for 12 weeks to evaluate its chondroprotective effect in vivo. By utilizing the HDHBL strategy to freeze the oriented polymer chains, different anisotropic hydrogels can be fabricated, expanding the potential application scope of these hydrogels, especially as substitutes for load‐bearing soft tissue.

## 2 Results and Discussion

### 2.1 Preparation and Characterization of Prestretched PNASC Hydrogels

In this study, we propose an HDHBL strategy that involves preloading PNASC‐based DMSO/water gels and inducing multiple hydrogen bond reconstruction through solvent exchange, ultimately leading to the formation of anisotropic hydrogels with exceptional properties (Scheme 1). First, PNASC DMSO/water gels were prepared with different initial monomer concentrations in a mixed solvent of DMSO and deionized water (7:3 v/v) through photo‐initiated free radical polymerization (denoted as PNASC‐X, where X represents the volume percentage concentration of the NASC monomer). Fourier transform infrared (FTIR) spectra of the as‐prepared PNASC DMSO/water gel (PNASC‐30%) and corresponding PNASC hydrogel after swelling equilibrium were shown in Figure S1 (Supporting Information). The FTIR spectrum of PNASC DMSO/water gel displayed characteristic bands of PNASC, including peaks at ν = 3388 cm^−1^ (m, NH), 3316 cm^−1^ (s, NH), 3203 cm^−1^ (m, NH), 1659 cm^−1^ (vs, C = O), and 1550 cm^−1^ (vs, NH). Notably, the absence of the C = C band at ≈ 1624 cm^−1^ in the FITR spectrum suggested the occurrence of the polymerization reaction of NASC monomers.^[^ ^29^ ^]^ Subsequently, the tensile properties of the PNASC DMSO/water gels were assessed (Figure S2, Supporting Information). Based on the mechanical results and considering that the higher hydrogen bonding density is essential to “lock” the oriented molecular chain conformation, we opted to utilize the PNASC DMSO/water gel with a higher concentration (30%) for constructing the anisotropic hydrogel through HDHBL strategy in subsequent experiments. As shown in Figure S1 (Supporting Information), a red shift of the characteristic band of C = O from 1659 cm^−1^ in PNASC DMSO/water gel to 1648 cm^−1^ in PNASC hydrogel was observed, revealing stronger hydrogen bonding interactions formed in PNASC hydrogel after solvent exchange.

**Figure 1:**
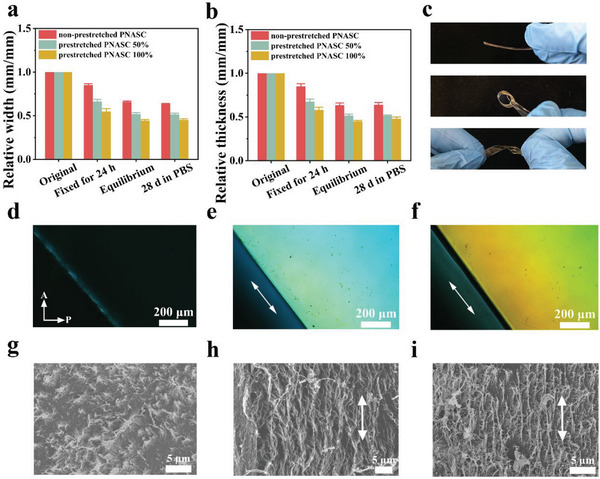
Evolution of a) relative width and b) thickness of the PNASC and prestretched PNASC hydrogels during the prestretching and solvent exchange process as well as after being immersed in PBS for 28 d. Data are presented as the mean ± SD (n = 4). c) Prestretched PNASC 50% hydrogel can withstand its weight and deformations including rolling and twisting (from top to bottom). d–f) POM images of the non‐prestretched PNASC (d), prestretched PNASC 50% (e) and prestretched PNASC 100% (f) (Scale bar: 200 µm). g–i) SEM images of the non‐prestretched PNASC (g), prestretched PNASC 50% (h) and prestretched PNASC 100% (i) (Scale bar: 5 µm). White arrows represent the prestretching direction.

Prestretching is the simplest preloading method, frequently employed to promote the orientation of molecular chains. To confirm the feasibility of the HDHBL strategy, PNASC‐30% DMSO/water gels were prestretched to different strains (50% and 100%) along the length direction, with both ends of each sample clamped and fixed. After exposure to room temperature and normal humidity for 24 h, the external load induced the alignment of polymer chains along the prestretching direction, accompanied by partial dissociation and rearrangement of non‐covalent interactions (i.e., hydrogen bonds) among the abundant amide/urea side groups of the PNASC chains. Furthermore, upon exposure to room temperature and normal humidity for 24 h, the width and thickness of the PNASC‐30% gel decreased, which further ensured a tightly packed arrangement of polymer chains. Subsequently, to “lock” the orientation of the polymer chains, the prestretched PNASC DMSO/water gels were immersed in deionized water for solvent exchange, inducing the reconstruction of hydrogen bonds and resulting in the formation of prestretched PNASC hydrogels (denoted as prestretched PNASC Y hydrogel, where Y represents the prestretching strain). After removing the hydrogen bond dissociation agent DMSO, the hydrogen bonds within the network were fully reconstructed, impeding the mobility of the PNASC molecular chains and effectively freezing their anisotropic conformations. For comparison, a non‐prestretched PNASC hydrogel sample was prepared by fixing its original length without undergoing the prestretching procedure. Next, to explore the effects of the HDHBL strategy on the prepared PNASC hydrogels, we monitored the size evolution of different samples during the prestretching and solvent exchange processes. As shown in **Figure** **1**a, b, compared to the original gels, both the width and thickness of the non‐prestretched and prestretched PNASC DMSO/water gels decreased after fixation for 24 hours and continued to decline until reaching swelling equilibrium. Notably, the width and thickness of the prestretched PNASC DMSO/water gels or hydrogels remained significantly lower than those of the non‐prestretched samples at the same stages, particularly when the gels were subjected to a larger prestretching strain. This shrinkage induced by interior stress promotes the aggregation of oriented polymer chains, resulting in a tightly packed network.^[^ ^28^ ^]^ The initial evidence was the lower equilibrium water contents (EWCs) of the prestretched PNASC hydrogels compared to the non‐prestretched hydrogels (Figure S3, Supporting Information). Despite this, it should be emphasized that the EWCs and water contact angles (WCAs) of the prestretched hydrogels remained above 40% and below 30°, respectively, indicating their inherent hydrophilicity and surface wettability (Figure S4, Supporting Information). Moreover, as presented in Figure 1a, b, the size of the prestretched PNASC hydrogels remained stable for 28 days in phosphate buffer saline (PBS) solution at 37 °C, confirming that PNASC chains were firmly locked by the reformed multiple hydrogen bond interactions. These results demonstrate the non‐swelling property of the prestretched PNASC hydrogels, a critical characteristic for tissue substitutes with long‐term stability.

**Figure 2:**
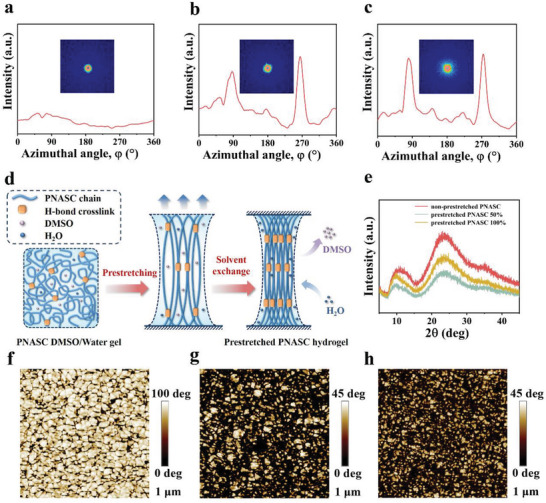
a‐c) SAXS patterns and scattering intensity‐corresponding azimuthal angle (*φ*) curves of the non‐prestretched PNASC (a), prestretched PNASC 50% b) and prestretched PNASC 100% hydrogels c). d) Schematic mechanism for high‐density hydrogen bond locking strategy for inducing and freezing the orientation of prestretched PNASC chains. e) XRD curves of the non‐prestretched and prestretched PNASC hydrogels. f–h) AFM phase images of the non‐prestretched PNASC (f), prestretched PNASC 50% (g) and prestretched PNASC 100% hydrogels (h) (Scale bar: 1 µm).

Subsequently, the microstructures of the prestretched PNASC hydrogels were evaluated. Based on the preliminary results, scattered patterns were observed as the light spot traversed through the non‐prestretched and prestretched PNASC hydrogels. The prestretched PNASC hydrogels exhibited significantly more anisotropic scattering compared to the non‐prestretched ones (Figure S5, Supporting Information). This finding was in accordance with previous reports on anisotropic hydrogels,^[^ ^21^ ^]^ and prompted us to further verify the anisotropic features of the prestretched PNASC hydrogels. As illustrated in Figure 1d–f, clear birefringence was evident in the prestretched PNASC hydrogels under polarizing optical microscopy (POM), whereas it was absent in the non‐prestretched samples, suggesting the formation of an anisotropic structure in the prestretched PNASC hydrogels. Furthermore, scanning electron microscopy (SEM) (Figure 1g–i) revealed that the freeze‐dried non‐prestretched PNASC hydrogel exhibited a randomly distributed architecture. In contrast, the freeze‐dried prestretched PNASC hydrogel displayed an anisotropic microstructure characterized by oriented microfibers along the prestretching direction. In addition, small‐angle X‐ray scanning (SAXS) patterns of the non‐prestretched and prestretched PNASC hydrogels (**Figure** **2**a–c) revealed that the orientation order parameter (*f*) of the prestretched PNASC hydrogels reached 0.9. This value was determined based on the scattering intensity plots against the corresponding azimuthal angle (*φ*) obtained from 2D SAXS patterns (Figure S6, Supporting Information). As illustrated in Figure 2d, the HDHBL strategy, which combines mechanical prestretching with solvent exchange, facilitated the formation of an anisotropic microstructure in the prestretched PNASC hydrogels. Specifically, the prestretching process aligns the randomly entangled molecular chains within the PNASC DMSO/water gel network along the direction of the external force. Upon immersion in water for solvent exchange, DMSO molecules are replaced by water molecules, leading to the reconstruction of high‐density hydrogen bond crosslinks between amide/urea side groups of the PNASC and significant contraction of the gel network. This results in the formation of densely arranged molecular chains and more compact hydrogen bond crosslinks, ultimately locking the anisotropic microstructure within the PNASC hydrogel network.

Next, we delved into the mechanism governing the locking of oriented polymer chains. X‐ray diffraction (XRD) analysis revealed the absence of crystalline peaks in both the non‐prestretched and prestretched PNASC hydrogels, indicating their amorphous nature (Figure 2e). The formation of disordered hydrogen bonding clusters in the PNASC hydrogel has been previously verified,^[^ ^29^ ^]^ which can lead to polymer chain aggregation and the development of microphase‐segregated structures. Atomic force microscope (AFM) phase images (Figure 2f–h) revealed microphase separation structures in both the non‐prestretched PNASC and prestretched PNASC hydrogels. These structures comprised regions with weak hydrogen bonding clusters (dark areas represent regions with relatively low modulus) and strong ones (bright areas represent regions with relatively high modulus).^[^ ^30^ ^]^ Notably, an increasing number of smaller hard microphases were formed in the prestretched PNASC hydrogels, especially in the prestretched PNASC 100% hydrogel. Therefore, the mechanical prestretching of the PNASC hydrogels resulted in not only an anisotropic microstructure but also a refined microphase, collectively enhancing the hydrogels’ ability to withstand external forces, akin to the grain refinement of crystalline polymers.^[^ ^31^, ^32^, ^33^, ^34^ ^]^

### 2.2 Mechanical Properties of the Anisotropic PNASC Hydrogels

The prominent microphase separation structure and dense network structure resulting from the HDHBL strategy endowed the anisotropic hydrogels (thickness ≈0.2 mm) with high rigidity, enabling them to withstand their weights and endure deformations, such as rolling and twisting (Figure 1c). This preliminary observation indicated their robust mechanical properties. Moreover, considering the strong anisotropic structure of the prestretched PNASC hydrogels, we anticipated their mechanical performance to also exhibit anisotropic characteristics. To confirm this hypothesis, we measured the mechanical properties of the prestretched PNASC hydrogels in different directions.

**Figure 3:**
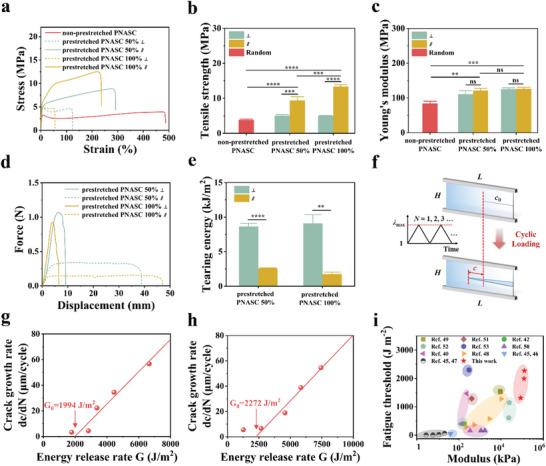
a) Tensile stress‐strain curves of the non‐prestretched and prestretched PNASC hydrogels obtained along random direction or directions parallel (∥) and perpendicular (⊥) to the prestretching direction and corresponding tensile strength b) and Young's modulus c). Data are presented as the mean ± SD (n = 3). P values were calculated using one‐way ANOVA followed by post hoc Bonferroni test. **P < 0.01, ***P < 0.001, ****P < 0.0001 and ns: no significance. d) Force‐displacement curves of the prestretched PNASC hydrogels obtained from the tearing tests along directions parallel (∥) or perpendicular (⊥) to the prestretching directions and corresponding tearing energy e). Data are presented as the mean ± SD (n = 3). P values were calculated using Student's t‐test. **P < 0.01, ****P < 0.0001. f) Experimental setup and geometry of the pure shear test (*L* > 2 H and initial notch length *c*_0_ = 2/5 *L*). During cyclic loading‐unloading tests, the maximum stretch ratio in each cycle was fixed at *λ*_max_, while the minimum stretch ratio was fixed at *λ* = 1.0. *c* is the length of crack propagation for notched samples and *N* is the number of cycles. Crack growth rate (*dc/dN*)‐energy release rate (*G*) fitting curves from fatigue‐resistance tests of the prestretched PNASC 50% g) and prestretched PNASC 100% h). i) Ashby plot of the fatigue threshold versus modulus for various hydrogels, i.e., single network polyacrylamide hydrogel,^[^ ^45^, ^46^ ^]^ double network polyacrylamide‐alginate hydrogel,^[^ ^45^, ^47^ ^]^ PVA‐based hydrogels (i.e., trained PVA hydrogel,^[^ ^40^ ^]^ annealed PVA hydrogel^[^ ^48^ ^]^ and 2D FC‐A PVA/GO hydrogel^[^ ^49^ ^]^), fiber‐matrix composites (i.e., PDMS composites,^[^ ^50^ ^]^ PDMS/PAAm composite,^[^ ^51^ ^]^ PAAm‐PAA‐Fe^3+^/PAA composite^[^ ^42^ ^]^ and GF‐PDMS composite^[^ ^52^ ^]^) and GelMA fiber hydrogel.^[^ ^53^ ^]^

As displayed in the tensile stress‐strain curves (**Figure** **3**a), both the non‐prestretched and prestretched PNASC hydrogels experienced five stages: elastic deformation, yielding, forced elastic deformation, strain hardening, and failure, along the orientation direction. It is worth noting that the strain softening behavior observed in the non‐prestretched PNASC hydrogel after yielding was absent in the tensile stress‐strain curves of the prestretched hydrogels along the direction parallel to the orientation axis, which implied that the prestretched PNASC hydrogels could bear heavier loads, especially along the oriented direction, than the non‐prestretched PNASC hydrogel even after yielding. Indeed, the prestretched PNASC hydrogels presented remarkably higher tensile strengths and Young's moduli in both parallel (∥) and perpendicular (⊥) directions to the orientation direction compared to the non‐prestretched samples (Figure 3b, c). In addition, the tensile properties of the prestretched PNASC hydrogels parallel to the orientation direction were significantly better than those in the perpendicular direction, partly because of the presence of aligned fibers resisting stretching. Specifically, the tensile strength of prestretched PNASC 100% parallel to the orientation direction (13.35 ± 0.59 MPa) was ≈ 2.7 times higher than that perpendicular to the orientation direction (5.01 ± 0.07 MPa), and over 3.4 times higher than that of non‐prestretched samples (3.83 ± 0.21 MPa). Moreover, Young's moduli of prestretched PNASC 50% and prestretched PNASC 100% (121.58 ± 6.49 MPa and 126.25 ± 4.33 MPa, respectively) were superior to that of non‐prestretched samples (83.84 ± 6.46 MPa). Additionally, the magnitude of prestretching strain significantly affects the mechanics of anisotropic hydrogels. In the direction parallel to the orientation axis, the prestretched PNASC 100%, subjected to a higher prestretching strain, possessed a tensile strength of 13.35 ± 0.59 MPa, significantly surpassing that of prestretched PNASC 50% (9.36 ± 1.10 MPa). This disparity can be attributed to the enhanced degree of alignment and the denser arrangement of polymer chains achieved under greater prestretching strain. More importantly, even after being immersed in PBS under 37 °C for 28 days, the mechanical properties of the prestretched PNASC hydrogels remained stable (Figure S7, Supporting Information).

In the subsequent experiment, a tearing test of the prestretched PNASC hydrogels in different directions was performed using trouser‐shaped samples. As illustrated in Figure 3d, e, the tearing energy of the prestretched PNASC hydrogels perpendicular to the orientation direction surpassed that in the parallel direction significantly. Specifically, for the prestretched PNASC 50% hydrogels, the tearing energy in the perpendicular direction reached 8.66 ± 0.46 kJ m^−2^, exceeding that in the parallel direction (2.60 ± 0.05 kJ m^−2^) by more than 3.3 times. In comparison, for the prestretched PNASC 100% hydrogels, the tearing energy in the perpendicular direction (9.10 ± 1.26 kJ m^−2^) was ≈ 5.2 times greater than that of parallel direction (1.76 ± 0.27 kJ m^−2^). This disparity is likely attributed to the anisotropic microstructure present in the prestretched PNASC hydrogels, which facilitated crack deflection in samples with initial precuts, thus inhibiting crack propagation along the original direction and averting the risk of catastrophic fracture. These findings underscore the potential of the prestretched PNASC hydrogels, with their remarkable and anisotropic mechanical properties, for applications requiring high tear resistance, such as load‐bearing tissue substitutes (e.g., meniscus, ligament, and tendon).

Fatigue‐fracture resistance under repeated biomechanical loads is crucial for many biological load‐bearing tissue replacements, particularly knee meniscal substitutes. Therefore, the fatigue resistance properties of both non‐prestretched and prestretched PNASC hydrogels were evaluated using cyclic pure shear tests of notched and unnotched prestretched PNASC hydrogel samples (Figure 3f), following the method employed by Suo et al..^[^ ^35^, ^36^, ^37^, ^38^, ^39^ ^]^ In tests with different maximum stretch ratios *λ*_max_ (exceeding 1.5), the crack propagation length (*c*) of the notched non‐prestretched PNASC hydrogel sample continued to increase with loading‐unloading cycles until the tensile stress approached zero (Figures S8 and S9, Supporting Information). Remarkably, for the notched prestretched PNASC hydrogels, *c* increased in the initial stage but reached a steady state after several cycles (Figures S10 and S11, Supporting Information). Similarly, the tensile stress of the notched prestretched PNASC hydrogels initially declined and then reached a plateau after 10000 loading‐unloading cycles (Figures S12 and S13, Supporting Information). Specifically, images of the notched prestretched PNASC hydrogels after 10000 cycles clearly demonstrated that crack deflection (Figure S14, Supporting Information), attributed to the presence of aligned microfibers and hydrogen bonding clusters in the anisotropic hydrogels. The crack growth rate at steady state, denoted as *dc/dN*, represents the slope of the *c* versus *N* plot, obtained after several cycles, where crack propagation reaches a steady state. Figure 3g, h and Figure S14, Supporting Information indicates that the rate of crack propagation in the prestretched PNASC hydrogels was significantly slower than that in the non‐prestretched PNASC hydrogel. The crack growth rate (*dc/dN*) as a function of energy release rate (*G*) was linearly fitted and the intercept of the linear relationship with the *G* axis was determined as the fatigue threshold *G*_0_. As plotted in Figure S15 (Supporting Information), the fatigue threshold of the non‐prestretched PNASC hydrogel was ≈ 1312 J m^−2^. In contrast, the fatigue thresholds of the prestretched PNASC 50% and prestretched PNASC 100% reached ≈1994 J m^−2^ (Figure 3g) and ≈2272 J m^−2^ (Figure 3h), respectively. These values were significantly higher than those of the non‐prestretched PNASC hydrogel and various other anisotropic hydrogels.^[^ ^40^, ^41^, ^42^, ^43^, ^44^ ^]^ To the best of our knowledge, achieving such a combination of ultrahigh stiffness and excellent fatigue resistance in anisotropic PNASC hydrogels is rare in other isotropic and anisotropic hydrogels. This is evident from the Ashby plot comparing fatigue threshold versus modulus for various hydrogels, including single network polyacrylamide hydrogel,^[^ ^45^, ^46^ ^]^ double network polyacrylamide‐alginate hydrogel,^[^ ^45^, ^47^ ^]^ PVA‐based hydrogels (i.e., trained PVA hydrogel,^[^ ^40^ ^]^ annealed PVA hydrogel,^[^ ^48^ ^]^ and 2D FC‐A PVA/GO hydrogel^[^ ^49^ ^]^), fiber‐matrix composites (such as PDMS composites,^[^ ^50^ ^]^ PDMS/PAAm composite,^[^ ^51^ ^]^ PAAm‐PAA‐Fe^3+^/PAA composite,^[^ ^42^ ^]^ and GF‐PDMS composite^[^ ^52^ ^]^) and GelMA fiber hydrogel^[^ ^53^ ^]^ (Figure 3i). Overall, the re‐establishment of high‐density hydrogen bonding clusters not only locks the aligned microfiber microstructure but also increases the network crosslinking density, thus inhibiting fatigue crack propagation and excessive deformation. Consequently, the prestretched PNASC hydrogel exhibited a combination of exceptional fatigue thresholds, an ultrahigh modulus, and superior strength, indicating greater potential as a load‐bearing soft tissue substitute with anisotropic features compared to other anti‐fatigue hydrogels.

**Figure 4:**
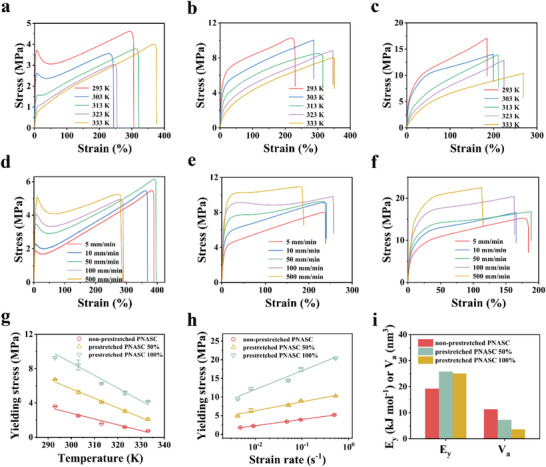
a–c) Tensile stress‐strain curves of the non‐prestretched PNASC (a), prestretched PNASC 50% (b), and prestretched PNASC 100% (c) hydrogels under different temperatures. d–f) Tensile stress‐strain curves of the non‐prestretched PNASC (d), prestretched PNASC 50% (e) and prestretched PNASC 100% (f) hydrogels under different strain rates. Variations of yielding stress as functions of temperature g) and strain rate h). Data are presented as the mean ± SD (n = 3). i) The activation energy of yielding (*E*_y_) and effective activation volume (*V*_a_) of the non‐prestretched and prestretched PNASC hydrogels.

The mechanical properties of polymeric materials are dependent on the movement of polymer chains. To further understand the effect of our HDHBL strategy on molecular chain motion and mechanics, the tensile properties of non‐prestretched and prestretched PNASC hydrogels were investigated under different temperatures and strain rates (**Figure** **4**a–f). Figure 4g reveals that the tensile yielding stress decreased linearly with an increase in temperature, whereas Figure 4h indicates that the increase in log(strain rate) was accompanied by an elevated yielding stress. These results can be described using the Eyring model for noncovalently crosslinked polymer systems:^[^ ^54^, ^55^ ^]^

(1) $$\dot{\varepsilon }\approx e^{-\left(E_{y}-0.5\sigma_{y}V_{a}\right)/\left(k_{b}T\right)}$$

where k_b_ is the Boltzmann constant, *T* is the absolute temperature, *σ*_y_, $\dot{\varepsilon }$, *E*_y_ and *V*_a_ are the tensile yielding stress, strain rate, activation energy of yielding and effective activation volume, respectively. The values of *E*_y_ and *V*_a_ of the prestretched PNASC 50% and prestretched PNASC 100% calculated by data fitting were 25.8 kJ mol^−1^ and 7.3 nm^3^, 25.1 kJ mol^−1^ and 3.6 nm^3^, respectively (Figure 4i). Compared to the non‐prestretched one (*E*_y_ = 19.3 kJ mol^−1^, *V*_a_ = 11.3 nm^3^), the prestretched PNASC hydrogels exhibited higher *E*_y_ and lower *V*_a_. *V*_a_ describes the size of PNASC chain segments involved in chain movement that results in yielding, while *E*_y_ represents the energy barrier that the PNASC chain segments must overcome to move under external force.^[^ ^54^, ^55^ ^]^ Therefore, the decreased *V*_a_ in the prestretched PNASC hydrogels implied diminished molecular mobility of PNASC chains, with the smallest *V*_a_ observed in the prestretched PNASC 100% corresponding to the lowest molecular mobility. In addition, the higher *E*_y_ in the prestretched PNASC samples hinted that dissociation of physical crosslinks (i.e., hydrogen bonding clusters) required higher tensile stress than the non‐prestretched sample, consistent with tensile test results.

To further confirm the reduction in molecular mobility of the polymer chains after prestretching, dynamic mechanical properties were determined through temperature sweep measurements. As displayed in Figure S16 (Supporting Information), the loss factor (tan *δ*) of non‐prestretched PNASC hydrogel exhibited a broad peak around 62 °C, corresponding to its glass transition temperature (*T*_g_). A comparison revealed that prestretched PNASC 50% and prestretched PNASC 100% presented higher *T*_g_ values, around 69 and 68 °C, respectively. The elevated *T*_g_ values suggest that the movement of the polymer chain segments in the prestretched PNASC hydrogels was more restricted compared to the PNASC hydrogel, potentially leading to stiffer mechanical performance. Importantly, the *T*_g_ values of both the non‐prestretched and prestretched PNASC hydrogels were significantly higher than room temperature, suggesting that all hydrogels were in a glassy state with forced elastic deformation observed in their tensile stress‐strain curves at room temperature.

In contrast to polymers with rigid backbones (such as cellulose), flexible polymer chains quickly return to their random coil conformation when the applied load is removed. This presents challenges in constructing anisotropic hydrogels using the prestretching method. Therefore, sufficiently strong intermolecular interactions are crucial for “locking” the oriented molecular chains to preserve the prestretching‐induced anisotropic microstructure in hydrogels composed of flexible polymer. Hydrogen bonds are ubiquitous interactions that play a pivotal role in maintaining the double helical structure of DNA. Despite this, the weaker hydrogen bond cannot “lock” the oriented microstructure. For instance, while PVA crystallization has been extensively employed for the fabrication of anisotropic hydrogels, achieving oriented microstructures in PVA gels solely through hydrogen bonding (without crystallization) remains unattainable, even after treatment with the drying in confined condition (DCC) method. Moreover, based on our previous investigation, although the PNAGA homopolymer hydrogel, crosslinked by multiple hydrogen bonds, exhibits non‐swelling behavior and excellent mechanical properties, preserving anisotropic characteristics induced by the prestretching treatment remains challenging. These cases prompted us to investigate the underlying factors contributing to the successful accomplishment of this challenge by the PNASC. Consequently, molecular dynamics simulations were performed to calculate the strength of intermolecular hydrogen bonding within PNASC chains. As reflected in Figure S17 (Supporting Information), in an aqueous environment, the calculated binding energy (−12.65 kcal mol^−1^) of the intermolecular hydrogen bond formed between PNASC chains closely resembles that of cellulose (−12.26 kcal mol^−1^) (Figure S18, Supporting Information), which has been reported to exhibit anisotropic hydrogel formation with precisely aligned hierarchical fibrous structures through DCC treatment.^[^ ^28^ ^]^ Comparatively, the intermolecular hydrogen‐bond binding energies of PVA (−4.98 kcal mol^−1^) (Figure S19, Supporting Information) and PNAGA (−7.83 kcal mol^−1^)^[^ ^29^ ^]^ are significantly lower than that of PNASC. These findings confirm that the strong intermolecular hydrogen bonding of PNASC in our HDHBL strategy effectively freezes the aligned molecular chains. This capability holds great promise for broadening the application range of flexible molecular chains in the fabrication of anisotropic hydrogels.

Motivated by the exciting outcomes mentioned above, we further explored the universality of incorporating the HDHBL strategy to construct other anisotropic hydrogels. We synthesized copolymers of the NASC monomer with the secondary monomers of N‐acryloyl glycinamide (NAGA), N‐acryloyl 2‐glycine (ACG), acrylamide (AM), and 2‐hydroxyethyl methacrylate (HEMA) to form P(NASC‐co‐NAGA), P(NASC‐co‐ACG), P(NASC‐co‐AM) and P(NASC‐co‐HEMA) copolymer DMSO/water gels. These PNASC‐based copolymer gels were initially prestretched to 100% strain and then underwent solvent exchange to yield prestretched P(NASC‐co‐NAGA), P(NASC‐co‐ACG), P(NASC‐co‐AM) and P(NASC‐co‐HEMA) hydrogels. As shown in Figure S20, Supporting Information, when compared to non‐prestretched PNASC‐based copolymer hydrogels, the tensile strengths and Young's moduli of the prestretched PNASC‐based copolymer hydrogels were increased considerably in the prestretching direction despite a decrease in extensibility. This trend was consistent with the effect of our HDHBL strategy for prestretched PNASC hydrogels. These findings demonstrate that PNASC is capable of serving as “high‐density hydrogen bond locks”, freezing the oriented chains of various polymers through copolymerization. This mechanism robustly maintains the mechanical anisotropy of flexible molecular chains.

### 2.3 Fabrication and Characterization of pl‐PNASC Meniscus Skeleton

**Figure 5:**
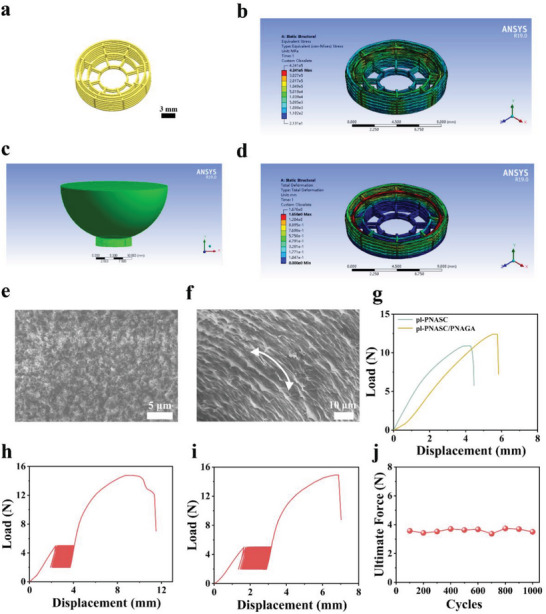
a) Model design diagram of the 3D printed PNASC meniscus construct (Scale bar: 3 mm). b–d) FEA results of the 3D printed PNASC meniscus construct during the preloading process. Finite element model (b) as well as corresponding stress field (c) and strain (d) field distributions of the 3D printed PNASC meniscus construct. e–f) SEM images of the PNASC (e) (Scale bar: 5 µm) and the pl‐PNASC meniscus scaffolds (f) (Scale bar: 10 µm). White arrow represents the circumferential direction. g) Suture pull‐out tests of the pl‐PNASC and pl‐PNASC/PNAGA meniscus scaffolds. Suture fatigue resistance of the pl‐PNASC h) and pl‐PNASC/PNAGA i) meniscus scaffolds with cyclic tensile loading‐unloading tests for 1000 cycles. j) Fatigue resistance of the pl‐PNASC/PNAGA meniscus scaffold with recording ultimate force of every 100 cycles.

We have demonstrated that we can create anisotropic PNASC‐based hydrogels with distinguished mechanical properties through mechanical preloading and HDHBL. This strategy was then extended to develop more complicated circumferentially anisotropic features for load‐bearing soft tissue substitutes (such as menisci). Scheme 1 illustrates the construction of a 3D‐printed anisotropic scaffold treated with mechanical preloading and solvent exchange‐induced hydrogen bond re‐establishment for meniscus replacement. To achieve a wedge‐shaped meniscus construct, we utilized a mixed solution comprising the NASC monomer, photoinitiator, UV absorber, DMSO, and water as the ink for digital light processing (DLP) 3D printing. Prior to the DLP 3D printing, a wedge‐shaped 3D meniscus model with a batch of circumferential and radial beams was meticulously designed, as presented in **Figure** **5**a and Figure S21a (Supporting Information). Following this design, a PNASC meniscus construct was established using DLP 3D printing (Figure S21b, Supporting Information).

To apply prestress to the obtained PNASC meniscus construct, we built a mechanical loading system comprising an electronic universal testing machine and a custom‐built compressive platen (Figure S22, Supporting Information). Before loading, a finite element analysis (FEA) was performed to determine an appropriate protocol by analyzing the strain and stress distributions in the PNASC meniscus construct during loading. As depicted in Figure 5b–d, the FEA results showed that a compressive load in the axial direction was applied to the PNASC meniscus construct, causing it to deform. Due to the wedge‐shaped design of the PNASC meniscus construct, the partial axial compressive stress was transferred into circumferential tensile stress through the circumferential and radial fibers, thus serving as a preloading step. According to the FEA, the PNASC meniscus construct could be compressed with an axial displacement of up to 1.6 mm, which accounts for 73% of its outer wall height. This displacement represents the compressive strain necessary for achieving maximum circumferential deformation without exceeding the bottom layer of the PNASC meniscus construct. During the mechanical loading experiment, incremental loads were applied by moving the compressive platen in the axial direction until reaching an equivalent axial displacement of 1.6 mm. Based on the mechanical loading experiment, the ultimate force applied to the PNASC meniscus construct was determined to be 25.9 N. This force was inputted into the FEA process to update the stress and strain field distributions accordingly. After 24 hours of mechanical loading, the PNASC construct compressed beneath the loading platen was immersed in deionized water for solvent exchange until swelling equilibrium was reached. This preloaded PNASC meniscus construct was named the pl‐PNASC meniscus scaffold (Figure S23a, Supporting Information). SEM images (Figure 5e, f) clearly demonstrated that the freeze‐dried pl‐PNASC meniscus scaffold exhibits a highly aligned microstructure in the circumferential direction, contrasting with the isotropic morphology observed in the freeze‐dried PNASC meniscus scaffold (DLP PNASC meniscus construct after reaching swelling equilibrium), manifesting the successful fabrication of circumferentially anisotropic meniscus scaffolds via mechanical preloading and the HDHBL strategy.

### 2.4 Preparation and Characterization of pl‐PNASC/PNAGA Meniscus Scaffolds

The resulting pl‐PNASC meniscus scaffold was utilized as a porous framework that emulated the collagen fiber network of native menisci to provide mechanical support. A flexible PNAGA high‐strength hydrogel was infused into the pl‐PNASC construct to replicate the water retention and cushioning functions of GAGs, resulting in a pl‐PNASC/PNAGA meniscus scaffold.^[^ ^56^, ^57^ ^]^ Benzophenone (BP), a photoresponsive initiator, was frequently utilized to integrate hydrogels with various materials through covalent bonds by abstracting hydrogen atoms from the C‐H bonds on the polymer chains to generate free radicals for grafting other macromolecular chains.^[^ ^58^, ^59^, ^60^ ^]^ In this study, we aimed to enhance the interfacial bonding between the pl‐PNASC skeleton and PNAGA hydrogel. Initially, the pl‐PNASC meniscus scaffold was treated with a BP solution and followed by a rinse with ethanol. Subsequently, a NAGA solution with a concentration of 30 wt% (containing a photo‐initiator) was perfused into the pl‐PNASC meniscus scaffold, and polymerization proceeded under UV irradiation. After that, the resulting scaffold was immersed in deionized water until swelling equilibrium was reached to obtain the pl‐PNASC/PNAGA meniscus scaffold (Figure S23b, Supporting Information). To evaluate the effect of BP treatment on the interfacial bonding between the two components, a 180° peeling test was performed. This test measured the interfacial strength between the PNAGA hydrogel and the prestretched PNASC 50% hydrogel, both with and without BP treatment. As demonstrated in Figure S24 (Supporting Information), the interfacial strength of prestretched PNASC 50%‐PNAGA hydrogel was significantly improved (from 125.5 ± 3.3 N m^−1^ to 849.4 ± 85.2 N m^−1^) after BP treatment. This indicates that the BP treatment considerably enhanced the interfacial bonding between the pl‐PNASC skeleton and the infused PNAGA hydrogel, facilitating effective load transmission between the two components.

To secure the proper positioning of the meniscus scaffolds within the knee joint, it is necessary to suture the scaffold edges to either the ligament or the joint capsule tissue. Therefore, achieving optimal suture resistance and fatigue resistance is essential for these scaffolds. As displayed in Figure 5g, the suture pull‐out strength of the pl‐PNASC/PNAGA meniscus scaffold measured 12.36 ± 0.20 N, surpassing that of the pl‐PNASC meniscus scaffold (10.53 ± 0.32 N) and significantly exceeding previously reported meniscus scaffolds.^[^ ^27^, ^61^ ^]^ This demonstrates the strong suture resistance of our meniscus scaffolds. Moreover, the pl‐PNASC/PNAGA meniscus scaffold possesses a higher suture pull‐out strength than the bare pl‐PNASC meniscus scaffold, which can be attributed to the synergistic effect of the high‐strength pl‐PNASC framework and the viscoelastic behavior of PNAGA cushion, thus providing mechanical support and energy absorbing functions. Moreover, the porous pl‐PNASC framework and infused PNAGA hydrogel are mechanically interlocked, which further facilitates effective load transfer. In addition, the suture fatigue resistance of the pl‐PNASC and pl‐PNASC/PNAGA meniscus scaffolds was assessed through cyclic tensile loading‐unloading tests for 1000 cycles. As presented in Figure 5h, i, the ultimate suture pull‐out strengths of both the pl‐PNASC and pl‐PNASC/PNAGA meniscus scaffolds remained high level (14.78 N and 14.92 N, respectively) even after the cyclic loading‐unloading tests, demonstrating their excellent suture fatigue resistance. These results implied that these meniscus scaffolds could retain precise positioning and structural integrity under both static and cyclic loading conditions. To assess the circumferential tensile resistance of the meniscus scaffolds, uniaxial tensile tests were performed along the circumferential direction on the PNASC (DLP PNASC meniscus construct after reaching swelling equilibrium), pl‐PNASC and pl‐PNASC/PNAGA meniscus scaffolds. As revealed in Figure S25 (Supporting Information), the pl‐PNASC/PNAGA meniscus scaffold presented a maximum load of 11.24 ± 0.79 N, which was higher than those of the pl‐PNASC meniscus scaffold (8.89 ± 0.61 N) and the PNASC meniscus scaffold (4.85 ± 0.53N). These results demonstrate that the pl‐PNASC/PNAGA network requires the highest circumferential tensile load to break. To further assess the fatigue resistance of the pl‐PNASC/PNAGA meniscus scaffold, successive cyclic compressive tests were performed without rest using a custom‐made device to simulate the biomechanical environment of the native meniscus, and the force variation with time was recorded. During the cyclic compressive test, a maximum strain of 12% was applied, consistent with the level experienced by the native meniscus under axial compressive stress within the knee joint, according to a previous study.^[^ ^62^ ^]^ As reflected in Figure 5j and Figure S26 (Supporting Information), the ultimate force at the maximum strain of 12% remained stable at ≈ 3.6 N, demonstrating the excellent fatigue resistance of the pl‐PNASC/PNAGA meniscus scaffold under repeated biomechanical loading.

Moreover, the in vitro cytotoxicity of the meniscus scaffolds was evaluated by examining the cell viability of L929 mouse fibroblast cells co‐cultured with the extract of the scaffolds for 1, 3, and 7 days. The cell viability results revealed that both the pl‐PNASC and pl‐PNASC/PNAGA meniscus scaffolds presented good cell compatibility without cytotoxicity (Figure S27, Supporting Information). The in vitro hemolysis property of the meniscus scaffolds was also assessed. Deionized water (DI water) and saline were set as positive and negative control, respectively. After 2 h co‐incubation, the supernatants of blood cells treated with saline, pl‐PNASC and pl‐PNASC/PNAGA meniscus scaffolds were colorless and transparent. In contrast, the supernatant in the positive control was red due to the rupture of red blood cells. Moreover, the hemolysis ratio suggested that the meniscus scaffolds possessed favorable blood compatibility with 1.16% for the pl‐PNASC scaffold and 0.04% for the pl‐PNASC/PNAGA scaffold, respectively (Figure S28, Supporting Information), which are much lower than clinical permission requirement (5%).

Additionally, the pl‐PNASC/PNAGA meniscus scaffold exhibited a higher EWC (61.0 ± 0.4%) than the pl‐PNASC meniscus scaffold (46.6 ± 0.7%) (Figure S29, Supporting Information), which resulted from the introduction of hydrophilic PNAGA hydrogel. As demonstrated in Figure S30 (Supporting Information), the in vitro stability results revealed that both the pl‐PNASC and pl‐PNASC/PNAGA meniscus scaffolds remained stable in PBS solution for 4 weeks, as evidenced by the absence of significant weight change. This stability is attributed to the non‐degradable and non‐swelling properties of the PNASC and PNAGA hydrogels, as extensively documented in our previous studies.^[^ ^14^, ^27^, ^29^, ^56^ ^]^

### 2.5 In Vivo Implantation of Meniscus Scaffolds

**Figure 6:**
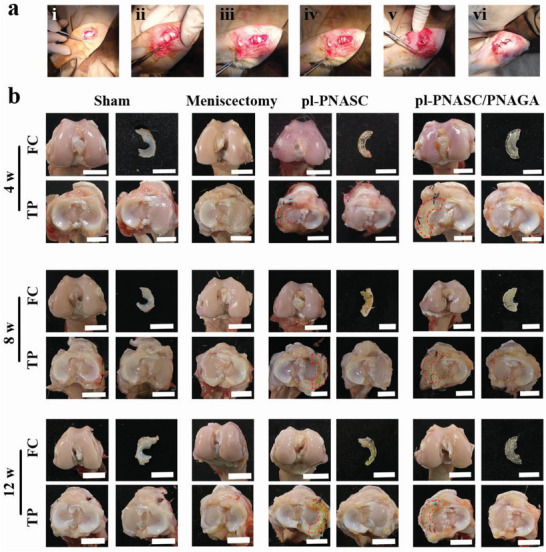
a) Implantation process of the pl‐PNASC and pl‐PNASC/PNAGA meniscus scaffolds in a rabbit model: (i) incising the skin tissue and the fascia tissue; (ii) severing the medial collateral ligament and opening the knee joint capsule; (iii) performing meniscectomy; (iv) implanting the meniscus scaffold; (v) suturing the medial collateral ligament and closing the knee joint capsule; (vi) suturing the skin tissue and the fascia tissue. b) Macroscopic observations of the femoral condyles (FCs) and the tibial plateaus (TPs) as well as the meniscal scaffolds at 4, 8, and 12 weeks (4 w, 8 w, and 12 w) post‐implantation. The rabbits with meniscus exposure only were designated as the sham group, while those subjected to meniscectomy alone comprised the meniscectomy group. In the experimental group, pl‐PNASC and pl‐PNASC/PNAGA meniscal implants were respectively transplanted into both knees of rabbits after total. The positions of the pl‐PNASC and pl‐PNASC/PNAGA meniscus scaffolds on the tibial plateaus were circled with red dotted lines (Scale bar: 1 cm).

Finally, the pl‐PNASC and pl‐PNASC/PNAGA meniscus scaffolds were implanted into the knee joints of rabbits for 4, 8, and 12 weeks post‐meniscectomy to assess their chondroprotective effects (**Figure** **6**a). All the procedures of animal experiments were conducted in compliance with the guidelines of the Council for the Purpose of Control and Supervision of Experiments on Animals, Government of China. The animal experiments were approved by the Animal Ethical Committee of Tianjin Institute of Medical and Pharmaceutical Science, China (IMPS‐EAEP‐H‐H2023001‐01).

After a certain period post‐implantation, initial gross observations were conducted with the macroscopic photographs demonstrated in Figure 6b. In the meniscectomy group, large cracks and abnormal abrasions were visible on the surfaces of the femoral condyle (FC) and tibial plateau (TP), with the condition cumulatively aggravating with prolonged postoperative time. After 12 weeks, certain areas of the FC cartilage surface exhibited severe erosion and delamination, leading to exposure of the subchondral bone due to increased intra‐articular contact stress after meniscectomy. By contrast, the implantation of the pl‐PNASC meniscus scaffold mitigated the erosion of the cartilage surfaces of the FCs and TPs, which was evidenced by minimal fissures and fibrous surfaces mainly confined to the superficial cartilages. Throughout the observation period, the pl‐PNASC meniscus scaffold remained in place, ensuring its ability to transfer loads effectively. However, the undesirable deformation of the pl‐PNASC meniscus scaffold reduced the contact area and increased the contact stress, resulting in scattered fissures after 8 and 12 weeks. In comparison, the FCs and TPs in the pl‐PNASC/PNAGA group demonstrated intact and glossy articular cartilage surfaces similar to those in the sham group. Even at 12 weeks after surgery, only minor cracks and light abrasion were visible on the surfaces of the FCs and TPs, indicating a normal cartilage state with no obvious degenerative changes. Furthermore, the pl‐PNASC/PNAGA meniscus scaffold remained un‐deformed and consistently in place throughout all observations. A histogram illustrating the postoperative coverage of the pl‐PNASC and pl‐PNASC/PNAGA meniscus scaffolds is presented in Figure S31 (Supporting Information). The coverage percentages of the postoperative pl‐PNASC and pl‐PNASC/PNAGA meniscus scaffolds resembled those of native menisci in the sham group across all time points. When considering the overall look, the macroscopic appearances of the cartilage surfaces of the FCs and TPs in the pl‐PNASC/PNAGA group were closest to those of the sham group, revealing that the pl‐PNASC skeleton and PNAGA hydrogel served as mechanical support and cushion to absorb energy, respectively. Therefore, their incorporation can protect articular cartilage from wear.

**Figure 7:**
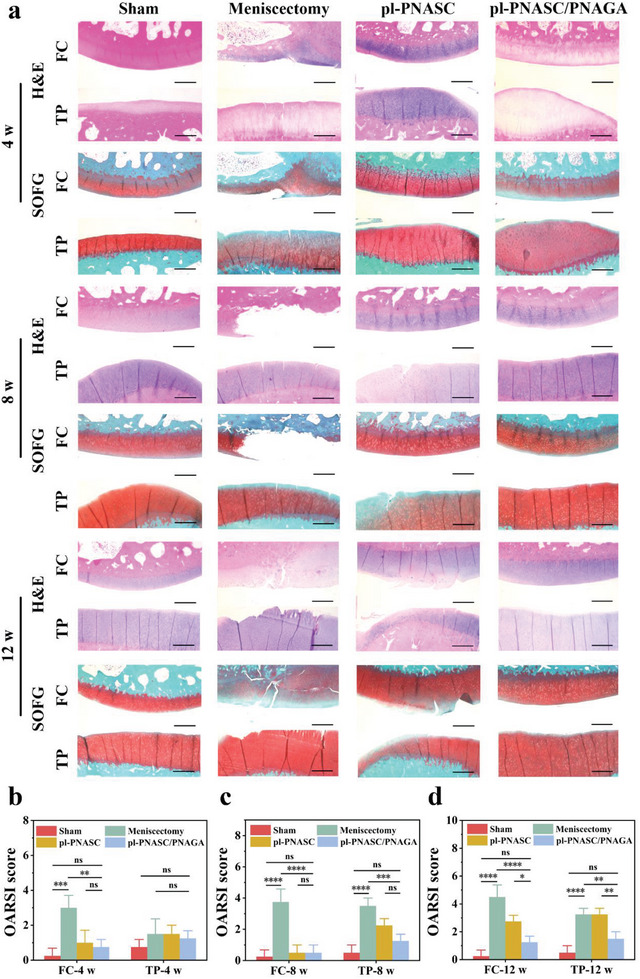
a) Histological evaluation of the femoral condyles (FCs) and the tibial plateaus (TPs) at 4, 8, and 12 weeks (4 w, 8 w, and 12 w) post‐implantation. H&E: hematoxylin and eosin staining, SOFG: safranin O‐fast green staining. The medial menisci replaced by the pl‐PNASC and pl‐PNASC/PNAGA meniscal implants were set as experimental groups, while the menisci of the sham group were exposed without excision and the menisci of the meniscectomy group were performed with meniscectomy (Scale bar: 500 µm). b–d) Osteoarthritis Research Society International (OARSI) scores assessing cartilage degeneration at 4 w (b), 8 w (c) and 12 w (d). Data are presented as the mean ± SD (n = 4). P values were calculated using one‐way ANOVA followed by post hoc Bonferroni test. *P < 0.05, **P < 0.01, ***P < 0.001, ****P < 0.0001, ns: no significance.

Histological evaluations of the FCs and TPs in all groups were performed using hematoxylin and eosin (H&E) and safranin O‐fast green (SOFG) staining to assess the degeneration of articular cartilage (**Figure** **7**). As illustrated in Figure 7a, in the meniscectomy group, the matrix discontinuity in the superficial zone of the FC and TP was observed at 4 weeks, and vertical fissures were present on the cartilage surfaces after 8 weeks. Delamination and denudation of the surface matrix of the FCs and TPs indicated deteriorative erosion and cartilage degradation after 8 and 12 weeks. In contrast, the matrix of the FCs and TPs in the pl‐PNASC group displayed normal architecture and their cartilage was intact and continuous at 4 weeks. However, surface discontinuity and superficial fibrillation appeared on the cartilage surfaces of the FCs and TPs in the pl‐PNASC group at 8 and 12 weeks, which might have resulted from the excessive stiffness of the pl‐PNASC meniscus scaffold. In contrast, the FCs and TPs in the pl‐PNASC/PNAGA group consistently presented an intact superficial matrix and normal cartilage morphology at all time points, similar to those in the sham group. In addition, the Osteoarthritis Research Society International (OARSI) scoring was applied to grade cartilage degradation and progression of the osteoarthritic process. The OARSI scores of the FCs and TPs in the pl‐PNASC/PNAGA group, based on histological staining, were lower than those in the meniscectomy and pl‐PNASC groups, approaching those in the sham group (Figure 7b–d). These results corroborated that the pl‐PNASC/PNAGA meniscus scaffold could effectively protect the cartilage of the femoral condyle and tibial plateau from wear and tear, thus delaying the progression of osteoarthritis.

## 3 Conclusion

In summary, we proposed a novel high‐density hydrogen bond locking (HDHBL) strategy, which involves mechanically preloading a hydrogen bonding poly(N‐acryloylsemicarbazide) (PNASC) gel swollen with an aqueous solution containing hydrogen bond breaking agent (DMSO), and subsequent solvent exchange to induce multiple hydrogen bond reconstruction. This strategy effectively freezes the oriented molecular chains, thus maintaining the formed anisotropic microstructure of the hydrogel. Utilizing this strategy, we fabricated an anisotropic meniscal skeleton with circumferentially oriented PNASC fibers using digital light processing‐3D printing technology combined with the HDHBL approach. This skeleton was further enhanced by infusing a resilient PNAGA hydrogel to obtain the meniscus scaffold to replicate the microarchitecture and function of collagen fibers and glycosaminoglycan in native menisci. In vivo experiments conducted over 12 weeks in rabbit knee joints demonstrated that this anisotropic high‐strength hydrogel‐based meniscus scaffold effectively protected articular cartilage from abrasion and showed promise for long‐term meniscal replacement in preclinical applications. Furthermore, we demonstrated the versatility of the HDHBL strategy by fabricating various anisotropic polymer hydrogels through the simple copolymerization of different monomers with NASC, followed by preloading and solvent exchange. This approach offers a simplified and versatile method for an avenue to customizing high‐strength anisotropic hydrogels for diverse applications.

## Conflict of Interest

The authors declare no conflict of interest.

## Supporting information

Supporting Information

## Data Availability

Research data are not shared.
