# Correction to “A High‐Density Hydrogen Bond Locking Strategy for Constructing Anisotropic High‐Strength Hydrogel‐Based Meniscus Substitute”

**DOI:** 10.1002/advs.202412203

**Published:** 2024-10-22

Zhang Q, Yang X, Wang K, Xu Z, Liu W. A High‐Density Hydrogen Bond Locking Strategy for Constructing Anisotropic High‐Strength Hydrogel‐Based Meniscus Substitute. *Adv. Sci*. **2024**, *11*, 2310035.

https://doi.org/10.1002/advs.202310035

Recently, we discovered an error in the Supporting Information of the paper, which has no impact on the content and conclusion of the paper. In Figure S10 (Supporting Information), the crack propagation c‐cycle N curves of the notched prestretched poly(N‐acryloylsemicarbazide) (PNASC) 50% hydrogels were provided incorrectly. The wrong figure is the crack propagation c‐cycle N curves of the notched non‐prestretched PNASC hydrogels. The authors apologize for this oversight. The corrected Figure S10 is clearly marked with yellow in the corrected Supporting Information and is also provided below.

Corrected Figure S10

**Figure S10:**
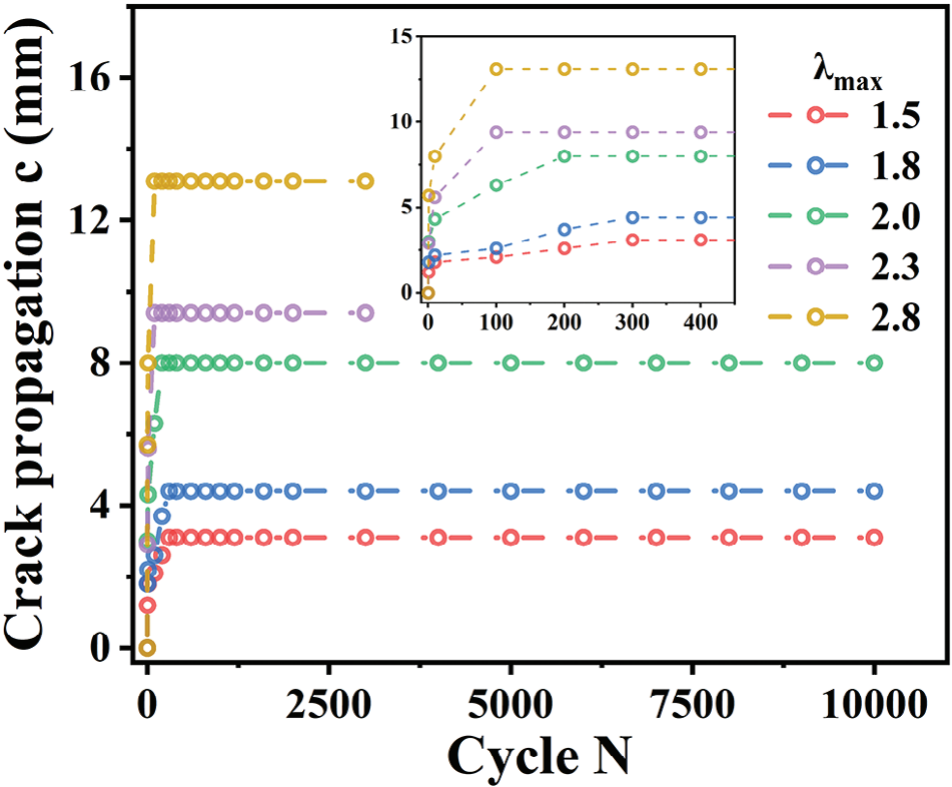
Crack propagation *c*‐cycle *N* curves of the notched prestretched PNASC 50% hydrogels upon cyclic loading‐unloading pure shear tests with different maximum stretch ratios (*λ*_max_).

We apologize for this error.

