# Supplementary material for: A High‐Density Hydrogen Bond Locking Strategy for Constructing Anisotropic High‐Strength Hydrogel‐Based Meniscus Substitute
Source: Adv Sci (Weinh). 2024 Mar 21;11(22):2310035. doi: 10.1002/advs.202310035 (PMC11165514; doi:10.1002/advs.202310035)
Supplement: Supplementary file 1 — Supporting Information [file ADVS-11-2310035-s001.pdf]

ADVANCED SCIENCE
Open Access

Supporting Information

for *Adv. Sci.*, DOI 10.1002/advs.202310035

A High-Density Hydrogen Bond Locking Strategy for Constructing Anisotropic High-Strength Hydrogel-Based Meniscus Substitute

Qian Zhang, Xuxuan Yang, Kuan Wang, Ziyang Xu and Wenguang Liu**

Supporting Information

A High-Density Hydrogen Bond Locking Strategy for Constructing Anisotropic High-Strength Hydrogel-Based Meniscus Substitute

Qian Zhang, Xuxuan Yang, Kuan Wang, Ziyang Xu, Wenguang Liu**

Q. Zhang, X. Yang, K. Wang, Z. Xu, Prof. W. Liu

School of Materials Science and Engineering, Tianjin Key Laboratory of Composite and Functional Materials, Tianjin University, Tianjin 300350, China

*Corresponding authors E-mail: xu_zy@tju.edu.cn; E-mail:wgliu@tju.edu.cn

Experimental Section

Materials

Semicarbazide hydrochloride (98%, Energy Chemical, Shanghai, China), glycinamide hydrochloride (98%, Macklin, Shanghai, China), glycine (≥98.5%, Alfa Aeser, Shanghai, China), acryloyl chloride (98%, Amethyst Chemicals, Beijing, China), ethyl alcohol (Yuanli Company, Tianjin, China), ethyl acetate (Yuanli Company, Tianjin, China), diethyl ether (Yuanli Company, Tianjin, China), potassium carbonate (Yuanli Company, Tianjin, China), 2-hydroxy-2-methyl-1-phenyl-1-propanone (IRGACURE 1173, 98%, Sigma-Aldrich), lithium phenyl(2,4,6-trimethylbenzoyl)phosphinate (98%, EFL Company, Suzhou, China), lemon yellow (EFL-UVAW-001, 98%, EFL Company, Suzhou, China), dimethyl sulfoxide (Yuanli Company, Tianjin, China), benzophenone (Macklin, Shanghai, China), acrylamide (AM) (TCI chemicals, Shanghai, China) and 2-hydroxyethyl methacrylate (HEMA) (Heowns, Tianjin, China) were used as received. L929 mouse fibroblast cells were provided by EK-Bioscience (Shanghai, China) and cell counting kit-8 was obtained from GlpBio (USA). N-acryloylsemicarbazide (NASC), N-acryloyl glycinamide (NAGA) and N-acryloyl 2-glycine (ACG) were synthesized as reported previously.[1-3] All other chemicals and solvents were analytical reagents.

Preparation of Prestretched PNASC Hydrogels

Poly (N-acryloylsemicarbazide) (PNASC) gels were prepared by photo-initiated free radical polymerization. Briefly, a certain mass of NASC monomer was dissolved in a mixed solvent of dimethyl sulfoxide (DMSO) and deionized water (7/3; v/v), and then 1 wt% of photo-initiator IRGACURE 1173 (relative to NASC) was added into above solution. The mixture was stirred under a nitrogen atmosphere until complete dissolution. Subsequently, the mixed solution was immediately poured into the mold and irradiated under 365 nm ultraviolet light (UV) for 40 min in a crosslinked oven (XL-1000 UV Crosslinker, Spectronics Corporation, USA) to obtain the PNASC gel impregnated with dimethyl sulfoxide (DMSO)/water (hereafter, abbreviated as PNASC DMSO/water gel), which named as PNASC-X (X=10%, 20%, 30%), where X represents the volume percentage concentration of NASC monomer. Unless otherwise

noted, PNASC-30% gel was used to further prepare non-prestretched and prestretched PNASC hydrogels. The obtained PNASC-30% gels were prestretched to certain strains (50% and 100%). Both ends of each sample were clamped and fixed using a homemade device. The prestretched PNASC DMSO/water gels were fixed for 24 h under room temperature and normal humidity. Finally, the whole device with the prestretched PNASC DMSO/water gels was immersed in deionized water and dialyzed for 5 days to completely remove DMSO and achieve the swelling equilibrium state. The prestretched PNASC hydrogel after swelling equilibrium was obtained and named prestretched PNASC Y (Y=50% and 100%, Y represents the prestretching strains of as-prepared PNASC-30% gels). For comparison, non-prestretched PNASC hydrogel samples were also prepared by dispensing with the prestretching procedure. The evolution of sample sizes (width and thickness) during the prestretching and solvent exchange process of the non-prestretched and prestretched PNASC hydrogels was recorded. Four specimens were measured for each group and the results were expressed as mean ± standard deviation.

Preparation of Prestretched PNASC-based Copolymer Hydrogels

PNASC-based copolymer gels were prepared by photo-initiated free radical copolymerization. Briefly, 0.15 g NASC and 0.15 g NAGA were dissolved in a 1 mL mixed solvent of DMSO and deionized water (7/3; v/v), and then 3 μL photo-initiator IRGACURE 1173 was added into above solution. The mixture was stirred under a nitrogen atmosphere until complete dissolution. Subsequently, the mixed solution was immediately poured into the mold and irradiated under 365 nm ultraviolet light (UV) for 40 min in the crosslinked oven to obtain the P(NASC-co-NAGA) gel. Similarly, 0.2 g NASC and 0.1 g ACG were copolymerized to obtain the P(NASC-co-ACG) gel, 0.2 g NASC, and 0.1 g AM were copolymerized to obtain the P(NASC-co-AM) gel, 0.25 g NASC and 0.05 g HEMA were copolymerized to obtain the P(NASC-co-HEMA) gel. As-prepared PNASC-based copolymer gels were prestretched to 100% strain and fixed for 24 h. Finally, the prestretched PNASC-based copolymer gels were immersed in deionized water and dialyzed for 5 days to completely remove DMSO and achieve the swelling equilibrium state. The prestretched PNASC-based copolymer hydrogels after

swelling equilibrium were obtained and named prestretched P(NASC-co-NAGA), prestretched P(NASC-co-ACG), prestretched P(NASC-co-AM) and prestretched P(NASC-co-HEMA), respectively. For comparison, non-prestretched PNASC-based copolymer hydrogel samples were also prepared by dispensing with the prestretching procedure and named non-prestretched P(NASC-co-NAGA), non-prestretched P(NASC-co-ACG), non-prestretched P(NASC-co-AM) and non-prestretched P(NASC-co-HEMA), respectively.

Digital Light Processing 3D Printing of PNASC Hydrogel-based Meniscus Construct

A PNASC hydrogel-based meniscus construct was fabricated by a digital light processing (DLP) apparatus (BP8600, EFL Company, China). Firstly, the DLP 3D printing ink solution was prepared by dissolving the monomer (NASC, monomer volume percentage concentration of 30%), photoinitiator (LAP, 5 mg/mL), and UV absorber (lemon yellow, 1 mg/mL) in a mixed solvent of DMSO and water (7/3; v/v). Subsequently, the prepared ink was carefully transferred into the ink tank. Prior to DLP 3D printing, a wedge-shaped 3D meniscus model with a batch of circumferential and radial fibers was developed by Pro/Engineer WildFire 5.0 software (Parametric Technology Corporation, USA) as our previous work.[4] Specifically, a 3D meniscus model with a diameter of 10 mm and a height of 2.2 mm was established and the obtained 3D printed PNASC meniscus construct was tailored after the preloading process. To mimic the microstructure of the native meniscus, we designed a wedge-shaped meniscus-mimic 3D model composed of a sequence of radial and circumferential fibers in our previous work.[4] In this study, we used the same model to construct a meniscus scaffold *via* DLP 3D printing technology. Briefly, to achieve the wedged shape, the number of fibers was decreased with the increase of the height of the model. In addition, to mimic the denser collagen fibers in the outer region than in the inner region of the native meniscus, a gradient decrease in fiber spacing was implemented from the central to the peripheral region of the model. The stereolithography (STL) files of the pre-designed 3D model were imported into the computer and printing parameters were set as layer thickness of 50 μm and curing time

of 5 s. During the printing process, the ink was exposed to blue light (405 nm, 10 mW/cm^2) to initiate the polymerization and form the cured hydrogel layer by layer. The obtained DLP PNASC construct was named as PNASC meniscus construct.

Fabrication of pl-PNASC and pl-PNASC/PNAGA Meniscus Scaffold

A hemispherical compressive platen with a diameter of 30 mm was fabricated with commercial photopolymer resin (Time80S, USA) by Form2 stereolithography (SLA) 3D printer (Formlabs, USA) for the mechanical loading system. Mechanical loading was applied on the PNASC meniscus construct using WDW-05 electronic universal testing machine (Jinan Time Shijin Instrument Corporation Limited, China) equipped with a custom-built compressive platen. Before loading, a finite element analysis (FEA) was performed to determine an appropriate protocol by analyzing local strain and stress fields in the PNASC meniscus construct during loading. A linear elastic finite element model with two contact components, the PNASC meniscus construct and the loading platen, was established by ANSYS software (ANSYS Incorporated, USA). The PNASC meniscus construct was modeled as linear elastic material with Young's modulus of 900 kPa, Poisson's ratio of 0.47 and tensile ultimate strength of 999.4 kPa. The Poisson' ratio of PNASC DMSO/water gel was estimated using the method as reported previously.[5] The loading platen was modeled as linear elastic resin with Young's modulus of 2460 MPa, a Poisson's ratio of 0.23 and tensile ultimate strength of 45.7 MPa (data from manufacturer). The stress and strain field distributions in the PNASC meniscus construct were analyzed with axial displacement up to 1.6 mm (73% of the outer wall height of the PNASC meniscus construct), which is a compressive strain to realize maximum circumferential deformation and not to exceed bottom layer of the PNASC meniscus construct. During the mechanical preloading treatment, increasing loads were applied by moving the compressive platen in the axial direction until an equivalent axial displacement (1.6 mm) was reached. According to the mechanical loading experiment, the ultimate force applied on the PNASC meniscus construct in the experiment was 25.9 N, and the force was imported into FEA process to refresh the stress and strain field distributions. After 24 h-mechanical loading, the PNASC construct compressed below the loading platen was immersed into deionized water and

dialyzed for 5 days to achieve swelling equilibrium. The preloading PNASC meniscus construct that underwent the above protocol was named as pl-PNASC meniscus scaffold.

Furthermore, the obtained pl-PNASC meniscus scaffold was treated with 10 wt% benzophenone (BP)-methanol solution for 10 min, and then the construct was washed with ethanol and air-dried under room temperature. Subsequently, the aqueous solution of NAGA (30 wt%) and 1 wt% of photo-initiator IRGACURE 1173 (relative to NAGA) was perfused into the pl-PNASC meniscus scaffold, and polymerization proceeded under 365 nm ultraviolet light irradiation. Specifically, a negative mold designed by computer-aided design technology with micro-CT data was used to accommodate the pl-PNASC framework and monomer solution of NAGA. The resultant scaffold was immersed into deionized water until swelling equilibrium to obtain the pl-PNASC/PNAGA meniscus scaffold.

Measurement of Fourier Transform Infrared (FTIR) Spectroscopy

Fourier transform infrared (FTIR) spectra of PNASC DMSO/water gel (PNASC-30%) and corresponding PNASC hydrogel after swelling equilibrium were analyzed and recorded with a scanning range of 4000-700 cm^{-1} and a resolution of 1 cm^{-1} by using a Nicolet 6700 Fourier Transform Spectrometer (Thermo Scientific, China).

Polarizing Optical Microscopic (POM) Observations

The birefringence of the non-prestretched and prestretched PNASC hydrogels was observed using a polarizing optical microscope (POM, Leica DM2700 M, Germany).

Scanning Electron Microscope (SEM) Observations

The surface morphologies of the freeze-dried non-prestretched and prestretched PNASC hydrogels as well as the freeze-dried PNASC (PNASC meniscus construct after swelling equilibrium) and the pl-PNASC meniscus scaffolds were sprayed with platinum, and then observed on s4800 scanning electron microscope (SEM, HITACHI, Japan) with an operating condition of 5 keV and 10 mA.

Small-Angle X-Ray Scattering (SAXS) Measurements

Small-angle X-ray scattering (SAXS) measurements were performed on NanoStar U SAXS System (Bruker, Germany) with an X-ray wavelength of 0.154 nm, and the

distance between the sample and the detector was 1050 mm. The non-prestretched and prestretched PNASC hydrogels (10 mm × 10 mm ×1 mm) were fixed on the clear aperture of the sample stage and then exposed to capture images. The scattering intensity-corresponding azimuthal angle (φ) curves were obtained from 2D SAXS patterns, and the orientation order parameter (f) was calculated according to Herman's orientation function.[6]

X-Ray Diffraction (XRD) Measurements

X-ray diffraction (XRD) patterns of the dried non-prestretched and prestretched PNASC hydrogels were recorded using a D8 Advanced X-ray diffractometer (Bruker, Germany). XRD data were obtained from 5° to 45° using Cu Kα radiation with a scanning rate of 3°/min at 40 kV and 40 mA.

Atomic Force Microscope (AFM) Observations

The surface topographies of the dried non-prestretched and prestretched PNASC hydrogels were observed on Dimension icon atomic force microscope (Bruker, USA) with the tapping mode. A scan size of 1 μm × 1 μm was used. Phase images of samples were processed and analyzed using NanoScope Analysis software (Bruker, USA).

Rheological Measurements

Rheological behaviors of the non-prestreched and prestretched PNASC hydrogels were measured using MCR 302 rheometer (Anton Paar, Austria) equipped with a 25 mm-diameter frosted plate as rotor and Peltier device for temperature control. All samples were cut into discs (about 25 mm in diameter and approximately 1 mm in thickness). Temperature sweep of the samples was conducted from 30 to 90 °C with a heating rate of 5 °C/min at a constant strain amplitude (0.05%) and a constant frequency (1 Hz).

Equilibrium Water Content (EWC) Measurements

The equilibrium water contents (EWCs) of the non-prestretched and prestretched PNASC hydrogels as well as the pl-PNASC and pl-PNASC/PNAGA meniscus scaffolds were measured as reported previously.[7,8] Three specimens were measured for each group and the results were expressed as mean ± standard deviation.

Water Contact Angle (WCA) Measurements

The surface water contact angles (WCAs) on the non-prestretched and prestretched

PNASC hydrogels were measured on a JC2000D contact angle measuring instrument (Zhongchen, China). 5 μL of deionized water droplet was dropped onto the surface of samples (10 mm × 10 mm × 1 mm) and images of the drops on the surface of samples were captured in a few seconds. The contact angles were calculated by the five-point fitting method. Three specimens were measured for each group and the results were expressed as mean ± standard deviation.

Mechanical Tests

The mechanical properties of the non-prestretched and prestretched PNASC hydrogels as well as the PNASC (PNASC meniscus construct after swelling equilibrium), the pl-PNASC and pl-PNASC/PNAGA meniscus scaffolds, were evaluated using Instron 2344 electronic universal testing machine (Instron, USA) equipped with a 500 N load cell in a water bath at room temperature unless otherwise stated. 30 mm × 4 mm × 1 mm rectangular samples of the non-prestretched and prestretched PNASC hydrogels were used for tensile tests. The uniaxial tensile tests were carried out at a stretching rate of 50 mm/min unless otherwise stated. The stress was obtained by dividing the force by the cross-sectional area of samples, and the strain was obtained by dividing the stretched length by the length of samples. Young's modulus of samples was calculated as the slope of the linear elastic region. Three specimens were measured for each group and the results were expressed as mean ± standard deviation. The cyclic compressive behavior of the pl-PNASC/PNAGA meniscus scaffold was evaluated using a custom-made device that recorded force changes over time during 1000 cycles of cyclic compressive loading-unloading tests at a rate of 10 mm/min, as previously reported.[9]

Tearing Tests

For the tearing test, two legs of the non-prestretched or the prestretched PNASC hydrogels with trouser shape were fixed on the clamps of the Instron 2344 and stretched with a loading rate of 50 mm/min. The tearing energy of the samples was calculated as follows (Equation 1):

$$\Gamma = \frac{2F_{\mathrm{ave}}}{d} \quad (1)$$

where F_{ave} is the average load during the stable tearing and d is the thickness of samples.

Three specimens were measured for each group and the results were expressed as mean ± standard deviation.

Fatigue Experiments

Fatigue experiments of the non-prestretched and prestretched PNASC hydrogels were performed using Instron 2344 in a water bath at room temperature. Unnotched and single-edge notched rectangular samples ($H_0 \times L \times t$) were used for the pure shear test. A precut of c_0 (two-fifths of L) from the edge was introduced into the single-edge notched sample. Cyclic tensile loading-unloading was carried out along the height direction at a nominal strain rate of 0.25 s^{-1}. The maximum stretch ratio in each cycle was fixed at λ_{max}, while the minimum stretch ratio was fixed at $\lambda = 1.0$. The length of crack propagation c for notched samples during cyclic loading-unloading tests was recorded as a function of the number of cycles N. The energy release rate G was calculated as follows (Equation 2):

$$G = W(\lambda_{max})H \tag{2}$$

where H is the initial distance between the two clamps (approximately 10 mm, $L > 2H$), and $W(\lambda_{max})$ is the elastic strain energy density of the unnotched sample at steady state under the corresponding λ_{max}. $W(\lambda_{max})$ was calculated from the integral area under the unloading curve at the 6000th cycle where the stress-stretch (σ-λ) curves reached the steady state. The crack growth rate at steady state dc/dN is the slope of c versus N plot taken after several cycles where the crack propagation reaches a steady state. For the prestretched PNASC hydrogels, the crack propagation length c increases at a stable rate from the initial stage and the dc/dN is taken after the 5th cycle and before the stasis; while the dc/dN of the non-prestretched PNASC hydrogel is taken after the 5th cycle. The crack growth rate dc/dN as a function of G was linearly fitted and the intercept of linear relation with the G axis was determined as fatigue threshold G_0.

180° Peeling Tests

The 180° peeling test was conducted at a stretching rate of 50 mm/min and room temperature using an Instron 2344 to evaluate the interfacial strength between prestretched PNASC hydrogel with/without BP treatment and PNAGA hydrogel under

swelling equilibrium state. The samples were fabricated by placing a tape between prestretched PNASC 50% and the PNAGA precursor solution to separate these two materials such that covalent bonding only existed in the overlapping region. The interfacial strength was calculated as twice the maximum peeling force divided by the width of the overlapping region.

Suture Pull-out Tests

To assess the suture resistance of the pl-PNASC and pl-PNASC/PNAGA meniscus scaffolds, the suture pull-out test was performed using 4-0 sutures with a stretching rate of 50 mm/min on Instron 2344 and the ultimate failure load was determined as suture pull-out strength. Note that the distance between the suture site and the peripheral rim of the meniscus scaffolds was 2 mm and surgical knots were made at the peripheral rim. Suture fatigue resistance was examined in water bath under room temperature by implementing loading-unloading tests with a load ranging from 2 to 5 N for 1000 cycles and finally stretching to failure.

Molecular Dynamics Simulations

The binding energy of hydrogen bond for PNASC, cellulose and PVA in water environment was studied by density functional theory (DFT), where all conformations of corresponding model compounds (double dimers) were optimized via Gaussian 16 C.02 at B3LYP functional[10,11] sand the def2-SVP[12,13] basis set level. The long-range van der Waals (vdW) interactions were handled by Grimme's DFT-D3 scheme.[14] Harmonic frequencies were performed at the same level to confirm that the structure corresponds to the minima on the potential energy surfaces. The binding energy was evaluated as follows (Equation 3):

$$BE = E(\text{double dimers}) - E(\text{dimer1}) - E(\text{dimer2}) \quad (3)$$

where the terms at right hand are the energies of the model compounds composed of double dimers, dimer1 and dimer2, respectively, at B3LYP-D3(BJ)/def2-QZVP[12,13] level of theory. All above calculations on solvent effect were implicitly incorporated by using the solvation model based on density (SMD) model.[15]

Cell Viability Evaluation of pl-PNASC and pl-PNASC/PNAGA Meniscus Scaffolds

The cytotoxicity of the pl-PNASC and pl-PNASC/PNAGA meniscus scaffolds was evaluated *via* cell counting kit-8 assay using L929 mouse fibroblast cells and the cell viability was calculated as reported previously.[4] Five specimens were measured for each group and the results were expressed as mean ± standard deviation.

Hemolysis Tests of pl-PNASC and pl-PNASC/PNAGA Meniscus Scaffolds

The hemolysis test of the pl-PNASC and pl-PNASC/PNAGA meniscus scaffolds was conducted and the hemolysis ratio was calculated according to a previously reported protocol.[16] Deionized water (DI water) and saline were set as positive and negative control, respectively. The supernatants of blood cells treated with different samples were photographed.

Evaluation of *In Vitro* Stability

The *in vitro* stability of the pl-PNASC and pl-PNASC/PNAGA meniscus scaffolds was investigated by soaking the samples in phosphate buffered saline (PBS, pH = 7.4) at 37 °C for 4 weeks as reported previously.[4] The weight change (%) of six specimens was measured for each group and the results were expressed as mean ± standard deviation.

***In Vivo* Implantation Experiment**

All the procedures of animal experiments were performed in accordance with the guidelines of the Council for the Purpose of Control and Supervision of Experiments on Animals, Government of China. The animal experiments were approved by the Animal Ethical Committee of Tianjin Institute of Medical and Pharmaceutical Science, China (IMPS-EAEP-H-H2023001-01). 42 male rabbits with weights of 2.5-3.0 kg were used in 4, 8, and 12-week experiments. All rabbits were divided randomly into 4 groups: sham group (n = 2 for each time point), meniscectomy group (n = 4 for each time point), pl-PNASC group (experimental group, n = 4 for each time point) and the pl-PNASC/PNAGA group (experimental group, n = 4 for each time point). The implantation procedure was conducted under sterile conditions as reported previously.[4] Before the surgery, both the pl-PNASC and pl-PNASC/PNAGA meniscus scaffolds were sterilized by 75% alcohol treatment overnight and UV irradiation for 1 h. The auricular intravenous injection of sodium pentobarbital solution with a dosage of 35

mg/kg was performed for anesthesia of all rabbits before the operation. After being fixed on the operating table, the legs of the rabbits were shaved and sterilized with iodine. Then, a total meniscectomy was carried out in both knees of the rabbits. Specifically, a 2 cm incision was made by dissecting the skin tissue and the fascia tissue. After that, the posterior horn of the medial meniscus was exposed by severing the medial collateral ligament. Next, the joint capsule was opened and the total meniscus was removed by dissecting the posterior and anterior horns along the junction of the medial meniscus and the tibial plateau. After the meniscectomy, the pl-PNASC or pl-PNASC/PNAGA meniscus scaffolds were grafted into the knee of rabbits. The anterior horn of the scaffolds was sutured to the ligament, while the posterior horn was connected to the joint capsule tissue. In the experimental groups, both legs underwent excision of the medial menisci and replacement with either pl-PNASC or pl-PNASC/PNAGA meniscal implants. In comparison, the menisci of the sham group were exposed without excision, while those of the meniscectomy group underwent surgical removal. Subsequently, the medial collateral ligament was connected and the joint capsule was closed. The fascia tissue and skin tissue were then sutured and the leg was immediately sterilized with alcohol pads. Finally, all rabbits were injected with penicillin for three days post-operation. In the following period, free activities of the rabbits in the cages were allowed. All rabbits were euthanized at 4, 8, and 12 weeks post-surgery and their femurs, tibias as well as meniscal implants were collected for further evaluation.

Macroscopic Observations and Histological Evaluation

Firstly, the femoral condyles (FCs), tibial plateaus (TPs) and the meniscus scaffolds of all groups were macroscopically observed and photographed. The coverage percentages (%) of postoperative pl-PNASC and pl-PNASC/PNAGA meniscus scaffolds were measured by Image J with the coverage percentage of the sham group as a standard (100%). After that, the aforementioned tissues were fixed in 4% paraformaldehyde for a duration of three days. The corresponding distal femur and proximal tibia were then decalcified using a 10% ethylenediamine tetraacetic acid (EDTA) solution over a period of twenty-eight days. Subsequently, all tissues underwent dehydration via graded

ethanol and were further embedded in paraffin. Finally, the decalcified tissues were stained with hematoxylin and eosin (H&E) as well as safranin O-fast green (SOFG) after being sectioned into the slices (thickness ~ 10 μm) to obtain the histological sections, which were observed and photographed using EVOS M5000 Inverted fluorescence microscope (Thermofisher, USA). Osteoarthritis Research Society International (OARSI) osteoarthritis cartilage histopathology assessment system was used to assess the cartilage degeneration of the FCs and TPs (a higher score corresponds to worse cartilage condition).[17] Blinded grading of the histological sections was performed by three independent evaluators according to the OARSI scoring system.

Statistical Analysis

All data are expressed as means ± standard deviations (SD). P values were calculated using Student's t-test for two-sample analyses. One-way analysis of variance (ANOVA) followed by post hoc Bonferroni test was performed to calculate the P values for multiple sample analyses. *$P < 0.05$, **$P < 0.01$, ***$P < 0.001$, ****$P < 0.0001$ were identified as statistical significance and ns represented no significance. GraphPad Prism 8.0 statistical software was used for statistical analysis of all data.

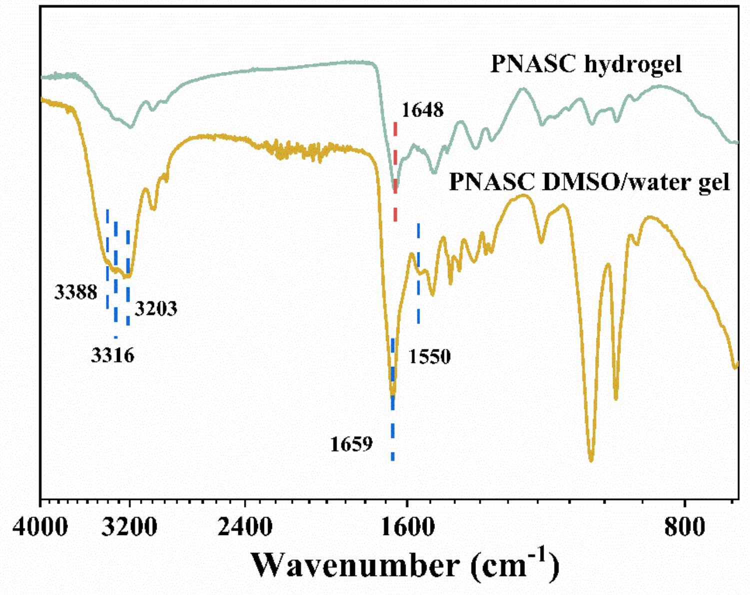

Figure S1. FTIR spectra of PNASC DMSO/water gel (PNASC-30%) and corresponding PNASC hydrogel after swelling equilibrium.

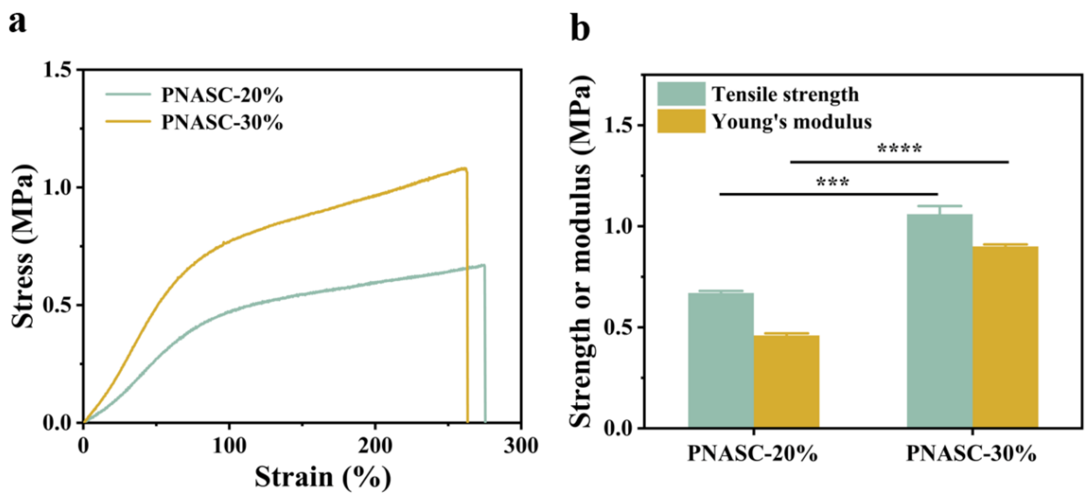

Figure S2. (a) Tensile stress-strain curves and (b) corresponding tensile strength as well as Young's modulus of the PNASC-20% and PNASC-30% DMSO/water gels. Note that PNASC-10% gel was too fragile to be stretched by the clamps. Data are presented as the mean ± SD ($n = 3$). P values were calculated using Student's t-test. ***$P < 0.001$. ****$P < 0.0001$.

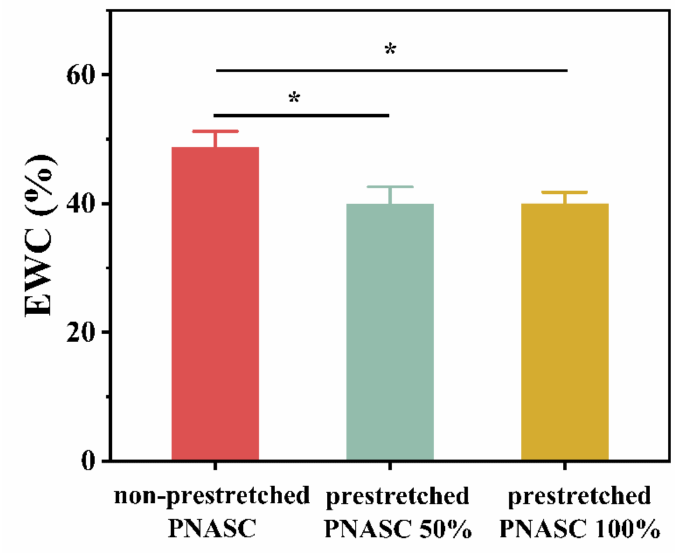

Figure S3. EWCs of the non-prestretched and prestretched PNASC hydrogels. Data are presented as the mean ± SD (n = 3). P values were calculated using one-way ANOVA followed by post hoc Bonferroni test. $^{*}P < 0.05$.

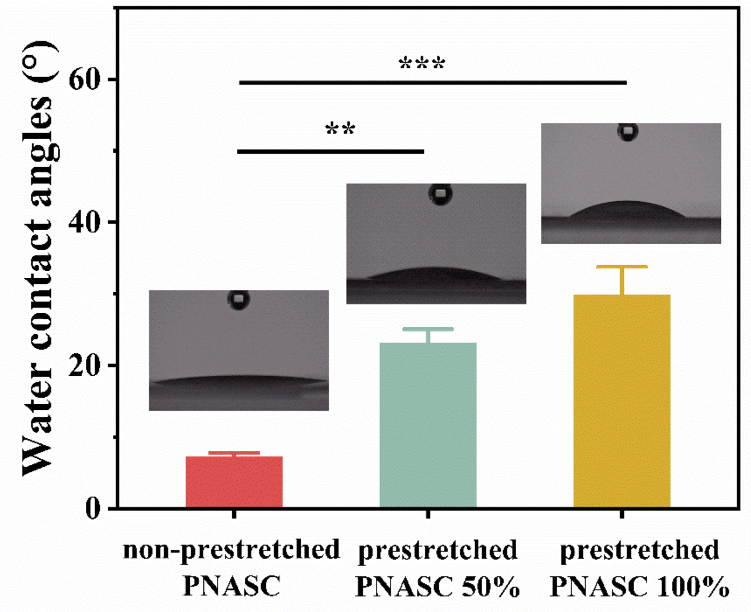

Figure S4. Water contact angles on non-prestretched and prestretched PNASC hydrogels. Data are presented as the mean ± SD ($n = 3$). P values were calculated using one-way ANOVA followed by post hoc Bonferroni test. $^{**}P < 0.01$, $^{***}P < 0.001$.

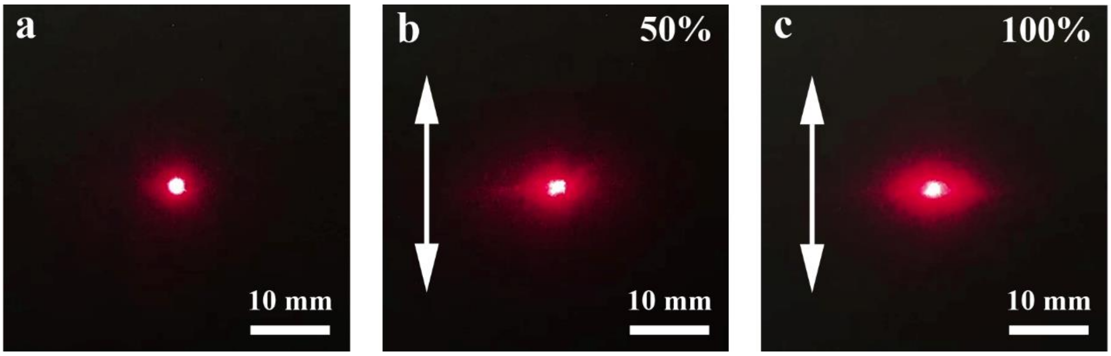

Figure S5. Photo images of the scattered light spot through the non-prestretched (a) and prestretched PNASC hydrogels with prestretching strains of 50% (b) and 100% (c). The results showed that light was scattered by the prestretched PNASC hydrogels. Note, the white arrows pointed in the prestretching direction (Scale bar: 10 mm).

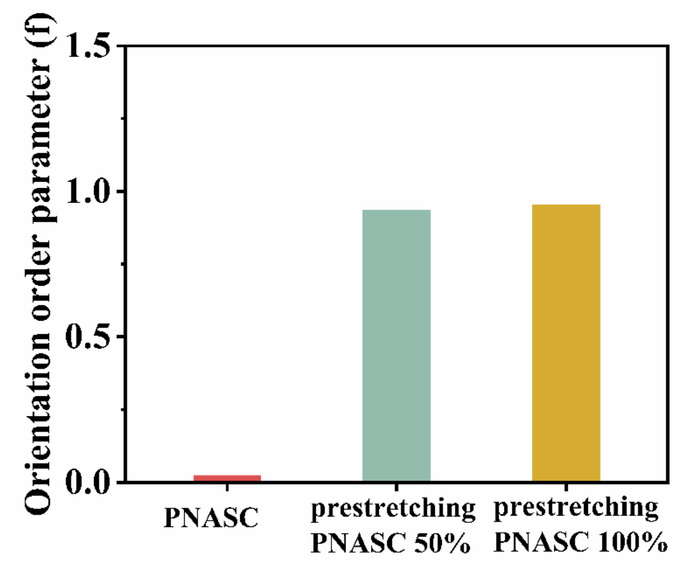

Figure S6. Orientation order parameter (*f*) of the non-prestretched and prestretched PNASC hydrogels calculated from the scattering intensity-corresponding azimuthal angle (φ) curves obtained from 2D SAXS patterns. The non-prestretched PNASC hydrogel possesses an f of 0.03, while f of the prestretched PNASC 50% and prestretched PNASC 100% hydrogels reaches up to 0.94 and 0.95, respectively.

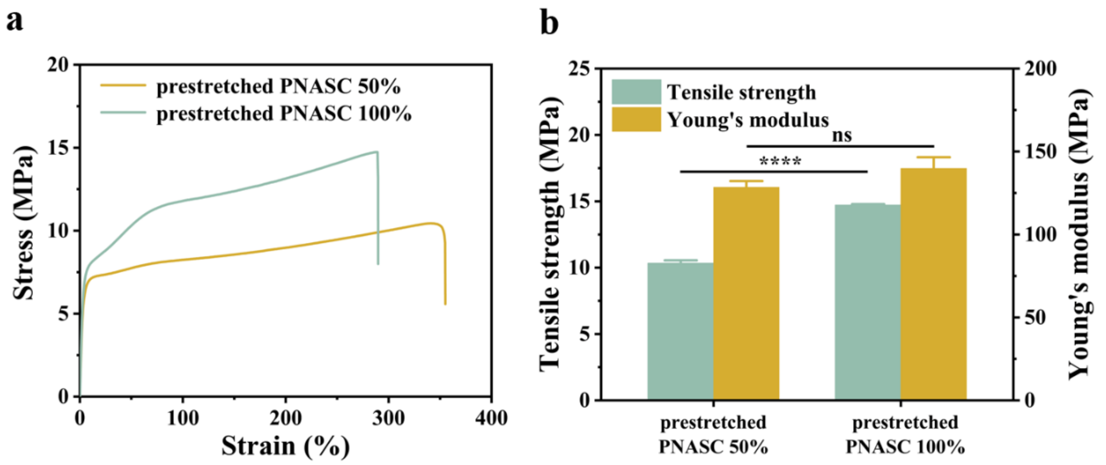

Figure S7. (a) Tensile stress-strain curves and (b) corresponding tensile strength as well as Young's modulus of the prestretched PNASC hydrogels immersed in phosphate buffer saline solution under 37 °C for 28 d. Data are presented as the mean ± SD (n = 3). P values were calculated using Student's t-test. ****$P < 0.0001$. ns: no significance.

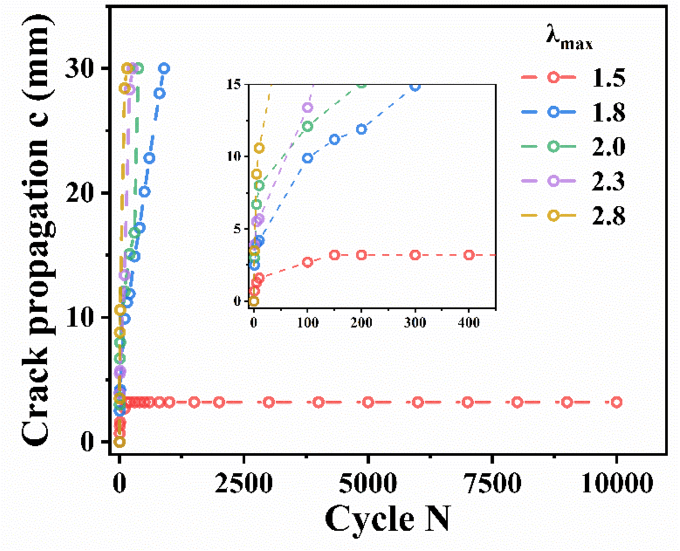

Figure S8. Crack propagation *c*-cycle *N* curves of the notched non-prestretched PNASC hydrogels upon cyclic loading-unloading pure shear tests with different maximum stretch ratios (λ_{max}).

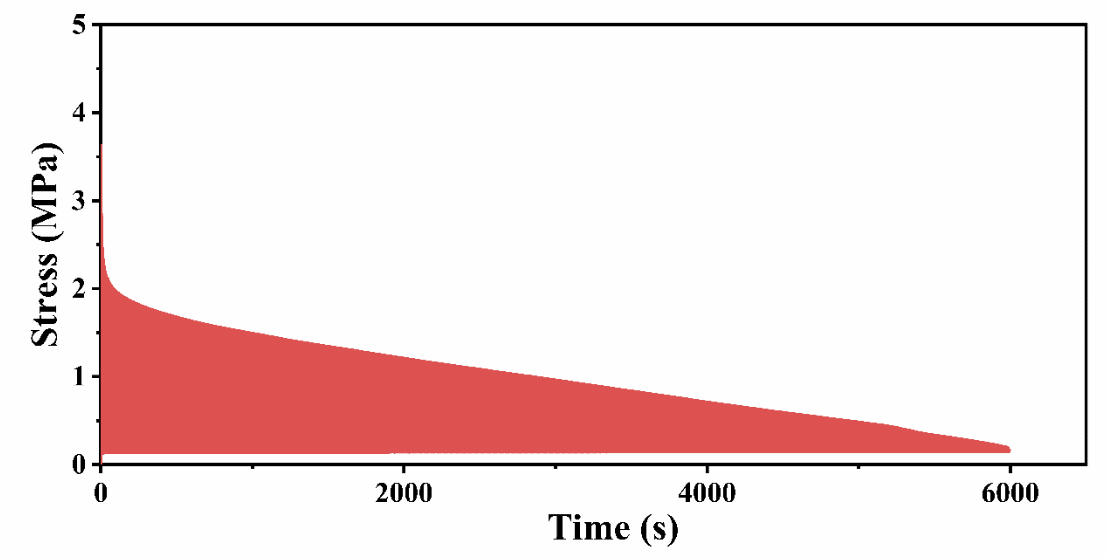

Figure S9. Stress-time curve of the notched non-prestretched PNASC hydrogel upon cyclic loading-unloading pure shear tests with a maximum stretch ratio of 1.8 and a minimum stretch ratio of 1.

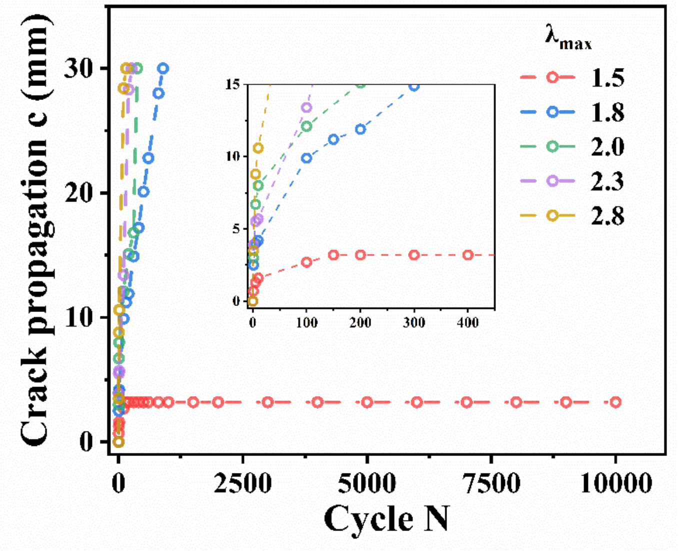

Figure S10. Crack propagation c-cycle N curves of the notched prestretched PNASC 50% hydrogels upon cyclic loading-unloading pure shear tests with different maximum stretch ratios (λ_{max}).

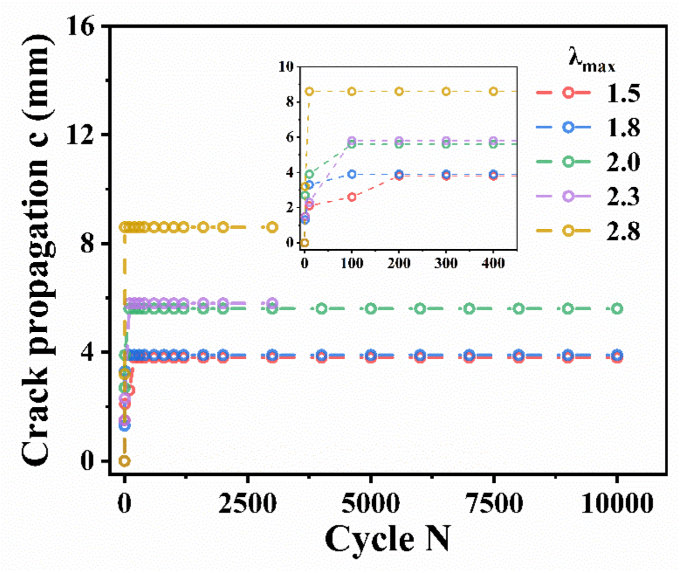

Figure S11. Crack propagation *c*-cycle *N* curves of the notched prestretched PNASC 100% hydrogels upon cyclic loading-unloading pure shear tests with different maximum stretch ratios (λ_{max}).

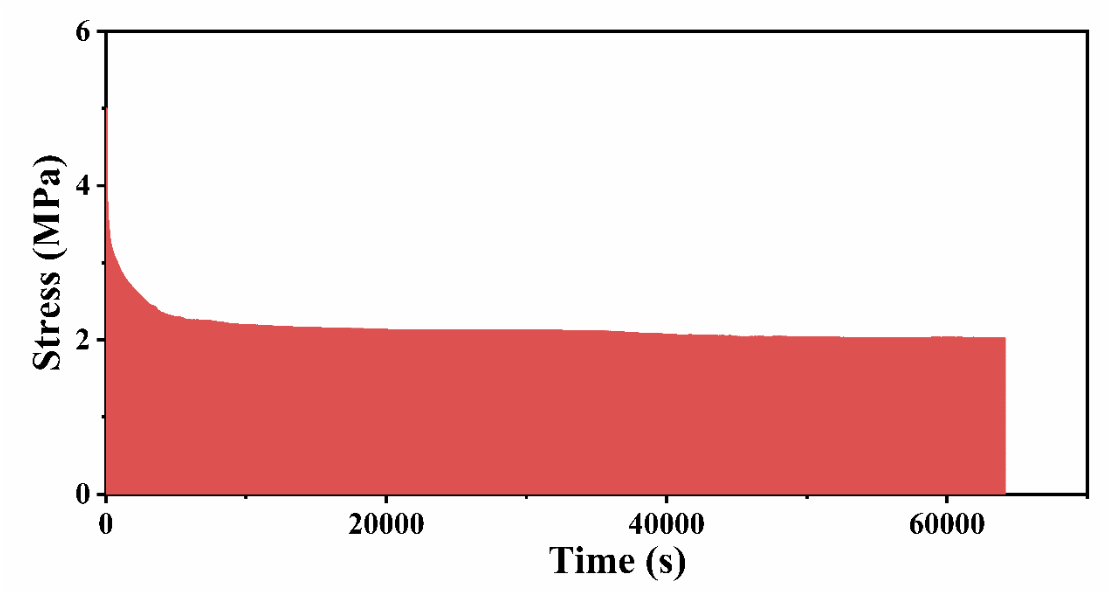

Figure S12. Stress-time curve of the notched prestretched PNASC 50% hydrogel upon cyclic loading-unloading pure shear tests with a maximum stretch ratio of 1.8 and a minimum stretch ratio of 1.

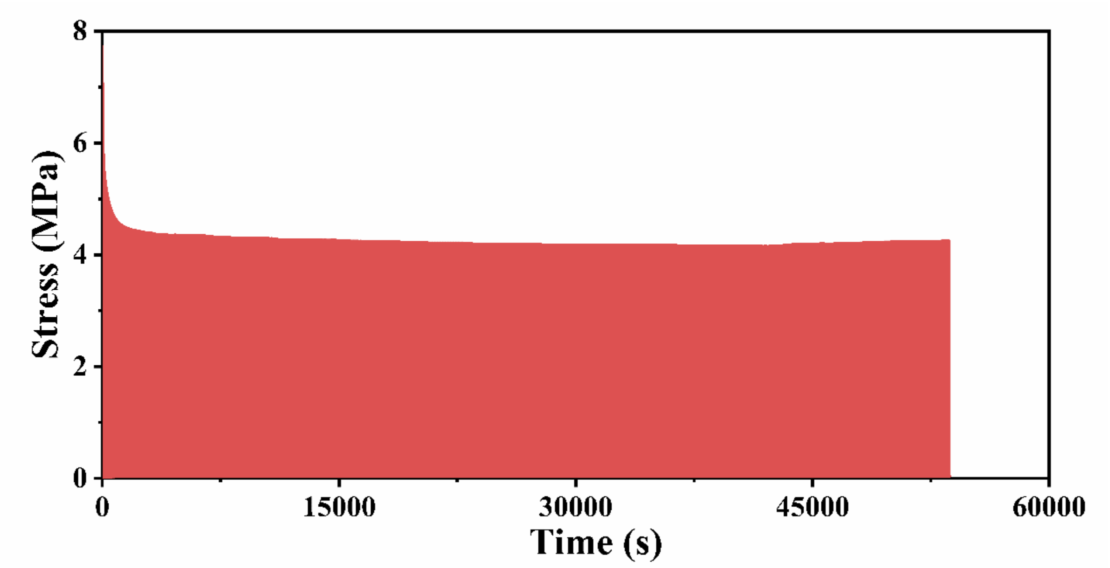

Figure S13. Stress-time curve of the notched prestretched PNASC 100% hydrogel upon cyclic loading-unloading pure shear tests with a maximum stretch ratio of 1.8 and a minimum stretch ratio of 1.

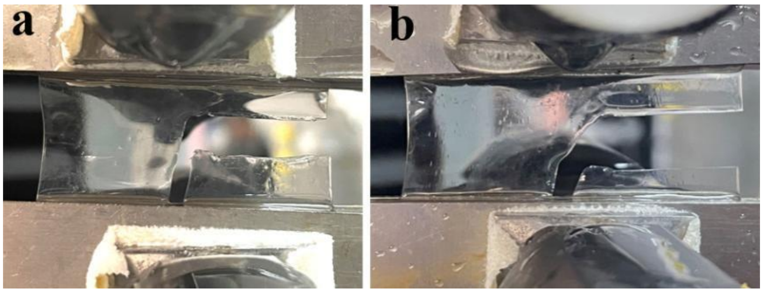

Figure S14. The pictures of the notched prestretched PNASC 50% (a) and PNASC 100% hydrogels (b) after ten thousand times of cyclic loading-unloading pure shear tests with a maximum stretch ratio of 1.8 and a minimum stretch ratio of 1.

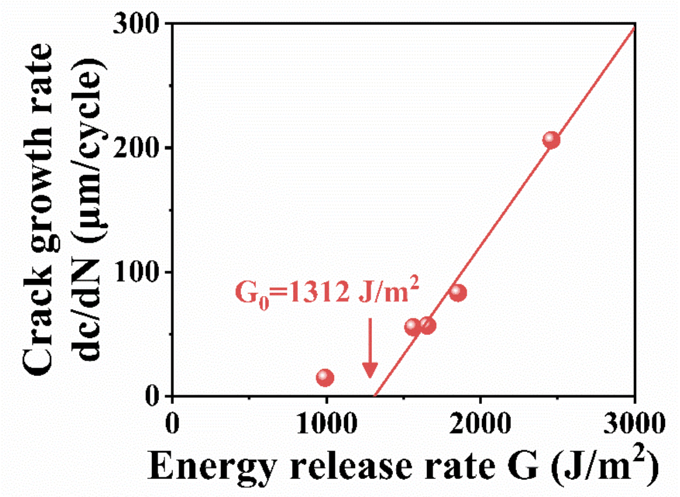

Figure S15. Crack growth rate (*dc/dN*)-energy release rate (*G*) fitting curve from fatigue-resistance test of the non-prestretched PNASC hydrogel.

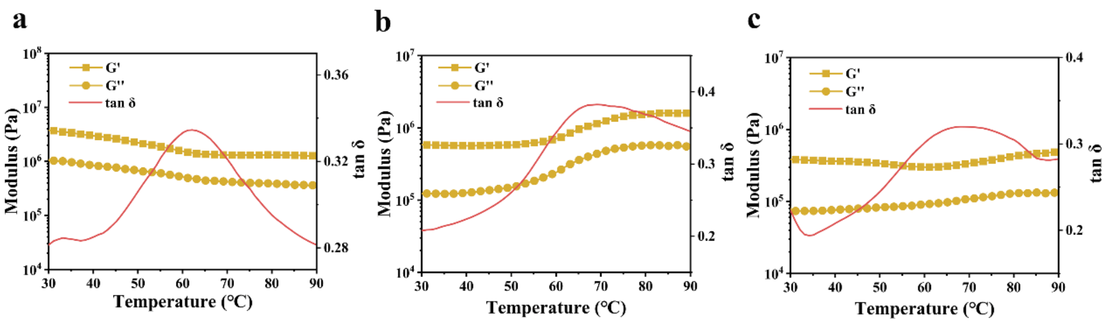

Figure S16. Rheological measurements with the temperature sweeping from 30 to 90 °C of the non-prestretched PNASC (a) and prestretched PNASC 50% (b) and prestretched PNASC 100% (c) hydrogels (G': storage modulus, G'': loss modulus, tan δ: loss factor).

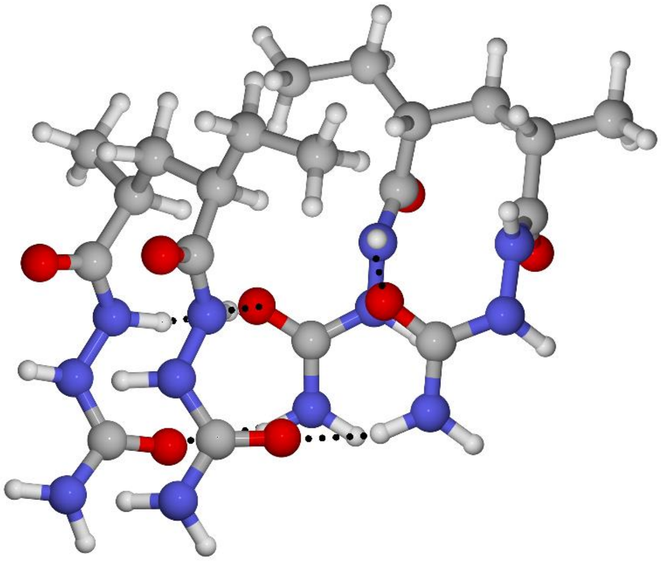

Figure S17. Conformation of PNASC model compound in water environment. The calculated binding energy of hydrogen bond formed between PNASC and PNASC is -12.65 kcal/mol.

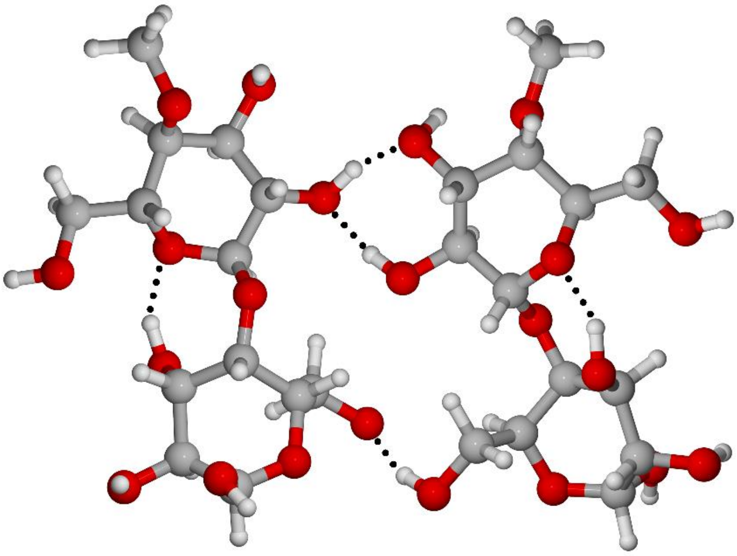

Figure S18. Conformation of cellulose model compound in water environment. The calculated binding energy of hydrogen bond formed between cellulose and cellulose is -12.26 kcal/mol.

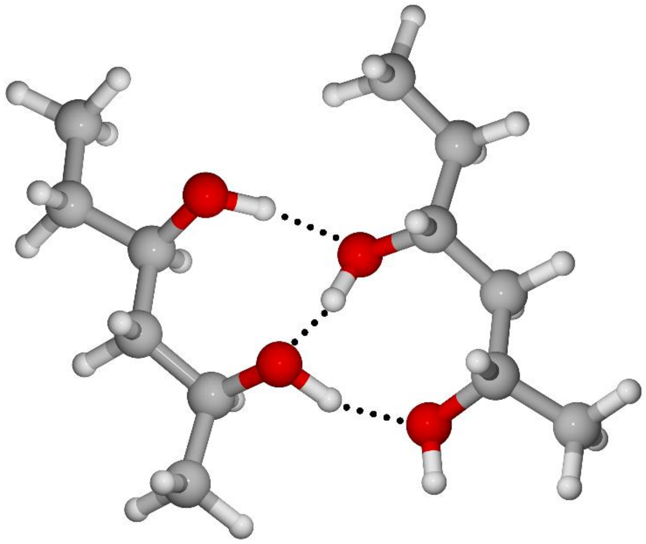

Figure S19. Conformation of PVA model compound in water environment. The calculated binding energy of hydrogen bond formed between PVA and PVA is -4.98 kcal/mol.

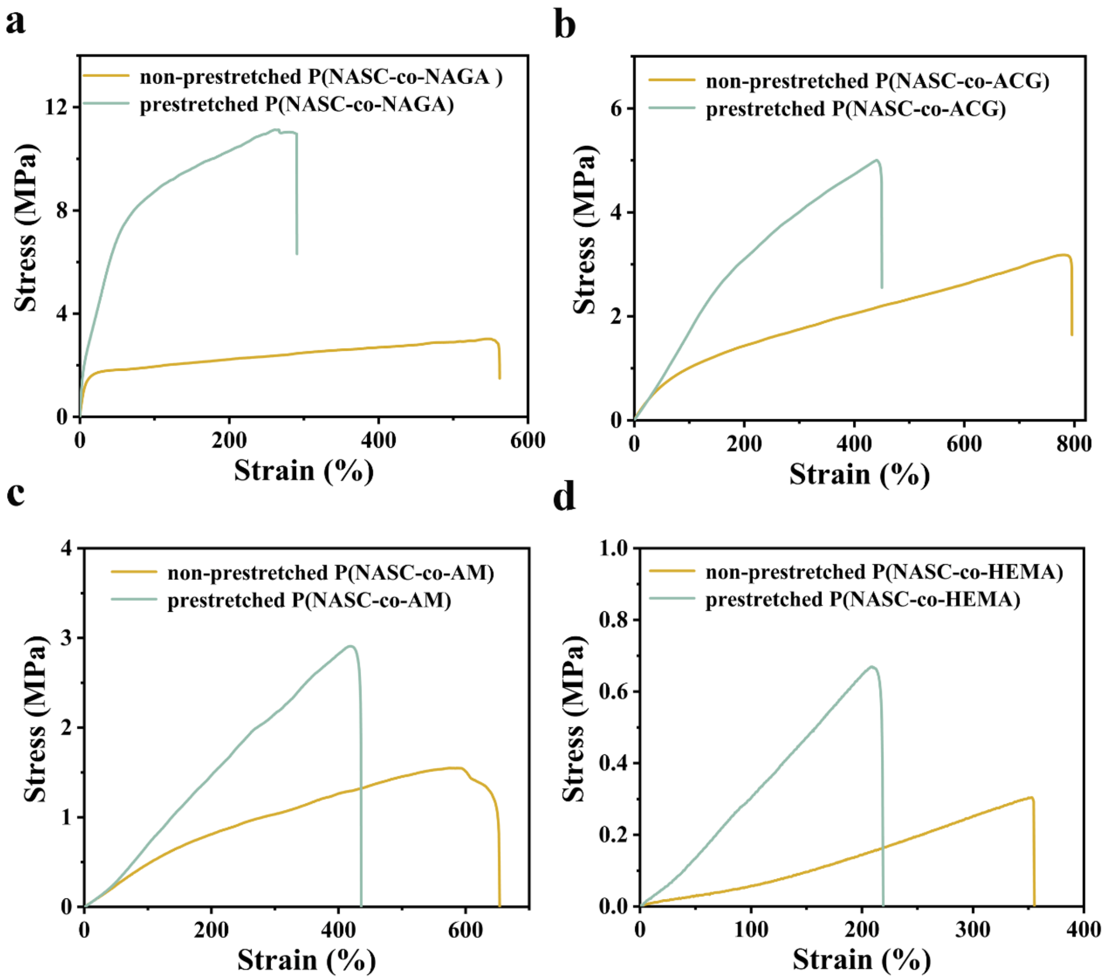

Figure S20. Tensile stress-strain curves of the non-prestretched and prestretched P(NASC-co-NAGA) (a), P(NASC-co-ACG) (b), P(NASC-co-AM) (c) and P(NASC-co-HEMA) (d) hydrogels.

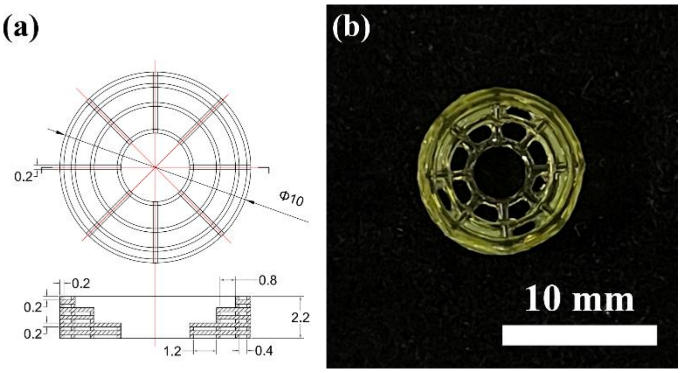

Figure S21. (a) Diagram of the meniscus model for DLP printing. (b) Macroscopic photograph of the DLP printed PNASC meniscus construct (Scale bar: 10 mm).

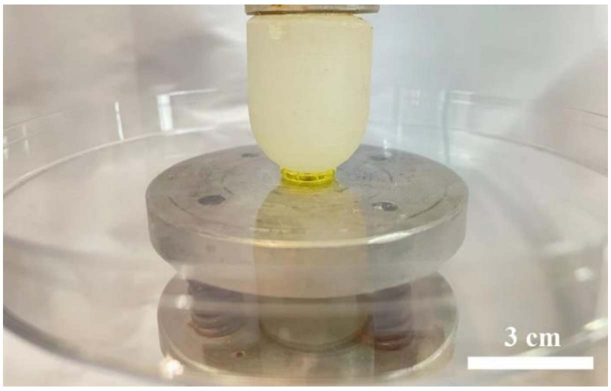

Figure S22. Mechanical loading system for fabricating the pl-PNASC meniscus skeleton.

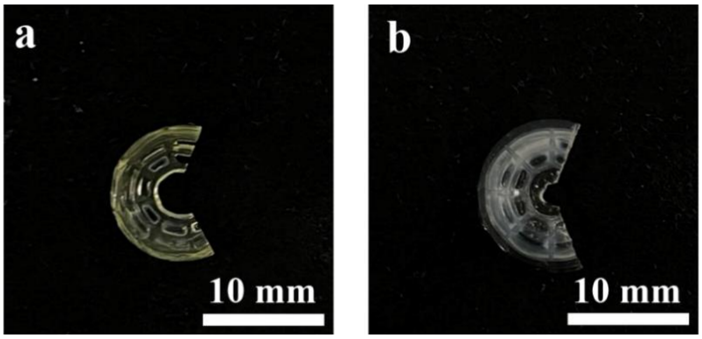

Figure S23. Macroscopic photographs of the pl-PNASC meniscus scaffold (a) and the pl-PNASC/PNAGA meniscus scaffold (b) (Scale bar: 10 mm).

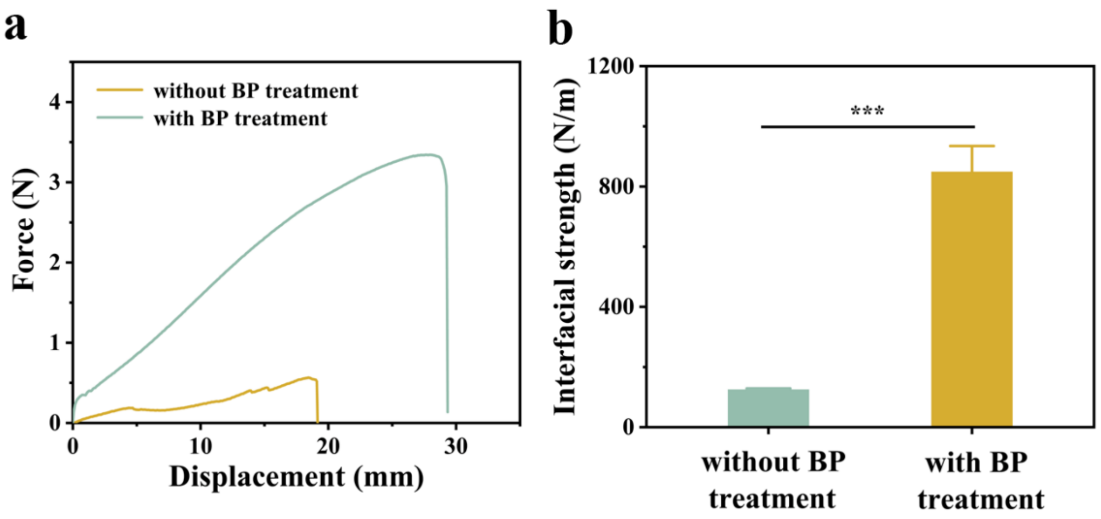

Figure S24. (a) Force-displacement curves of 180° peeling test and (b) interfacial strength between the PNAGA hydrogel and the prestretched PNASC-50% hydrogels with/without BP treatment. Data are presented as the mean ± SD (n = 3). P values were calculated using Student's t-test. ***P < 0.001.

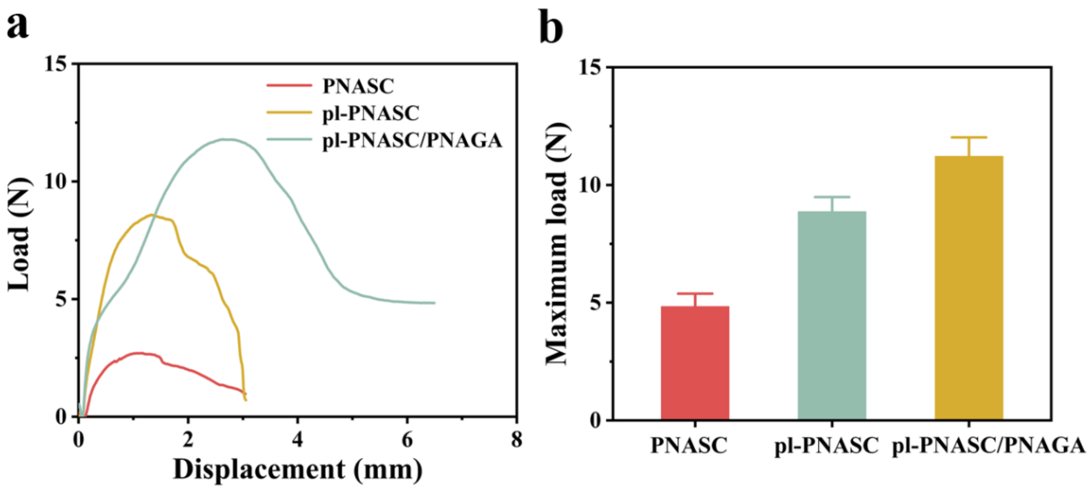

Figure S25. Circumferential tensile resistance of the PNASC, the pl-PNASC and pl-PNASC/PNAGA meniscus scaffolds. Load-displacement curves (a) and maximum loads (b) of the above meniscus scaffolds with uniaxial tensile tests along the circumferential direction.

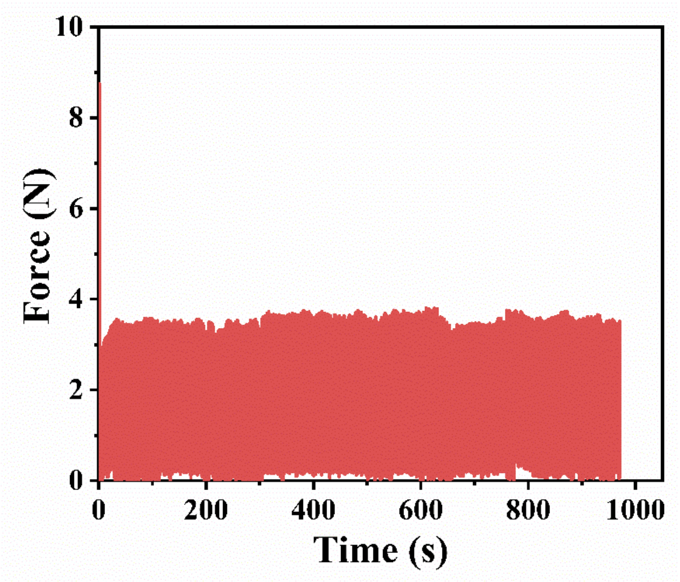

Figure S26. Fatigue resistance of the pl-PNASC/PNAGA meniscus scaffold was assessed by recording the force change with time under cyclic compressive loading-unloading tests for 1000 cycles.

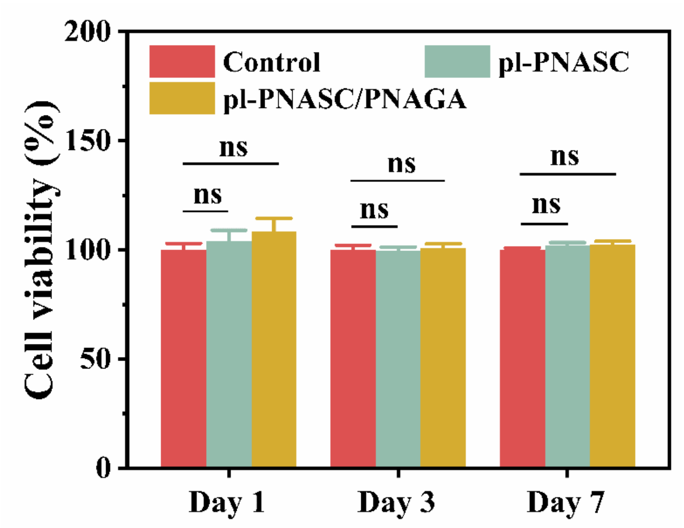

Figure S27. Cell viability of the L929 mouse fibroblast cells co-cultured with the extract of the pl-PNASC and pl-PNASC/PNAGA meniscus scaffolds for 1, 3, and 7 days. Data are presented as the mean ± SD (n = 5). P values were calculated using one-way ANOVA followed by post hoc Bonferroni test. ns: no significance.

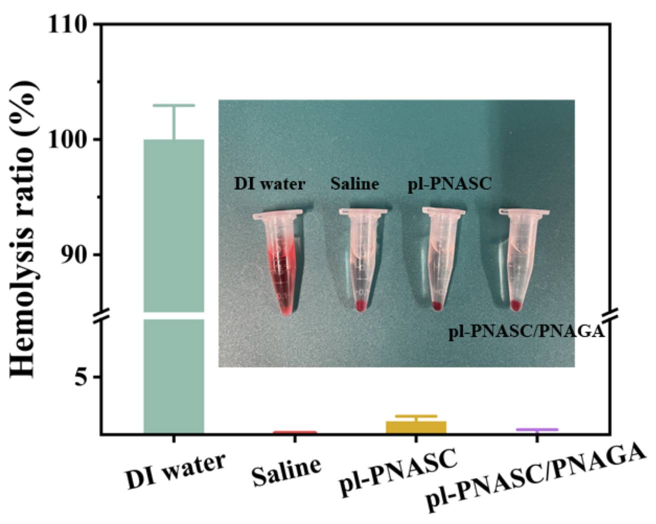

Figure S28. Hemolysis ratio of DI water, saline, pl-PNASC and pl-PNASC/PNAGA meniscus scaffolds and corresponding images of the supernatants of blood cells. Data are presented as the mean ± SD (n = 5).

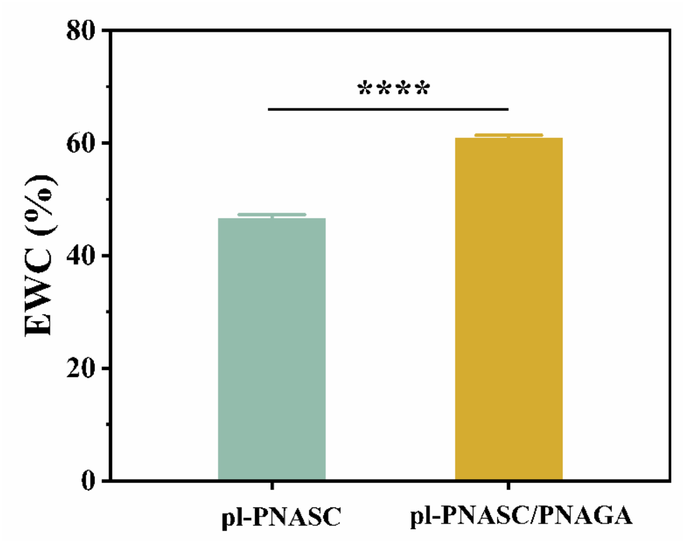

Figure S29. EWCs of the pl-PNASC and pl-PNASC/PNAGA meniscus scaffolds. Data are presented as the mean ± SD (n = 3). P values were calculated using Student's t-test. ****$P < 0.0001$.

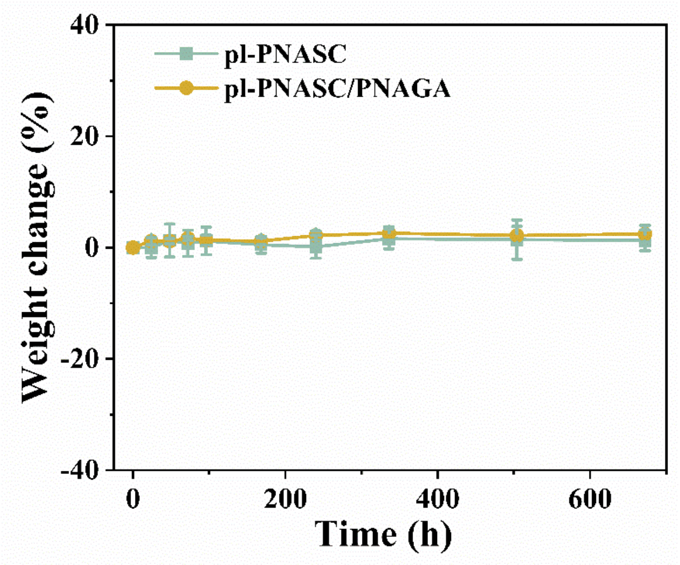

Figure S30. *In vitro* stability of the pl-PNASC and pl-PNASC/PNAGA meniscus scaffolds in PBS solution for 4 weeks. Data are presented as the mean ± SD (n=6).

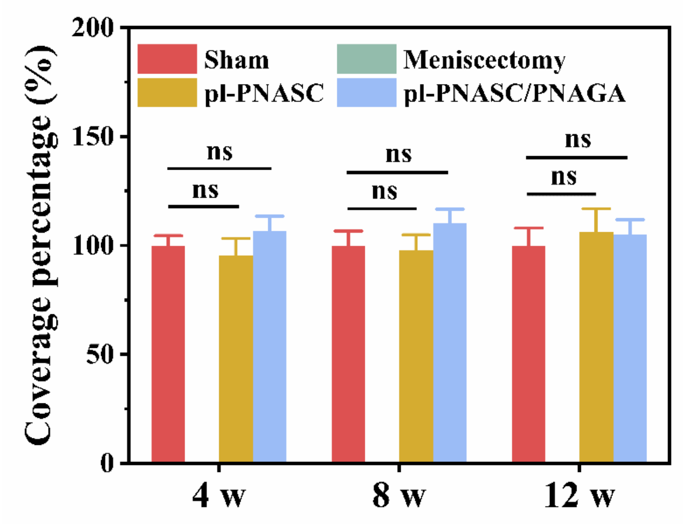

Figure S31. Coverage percentages of native menisci, the pl-PNASC and pl-PNASC/PNAGA meniscus scaffolds at 4, 8, and 12 weeks post-operation. Data are presented as the mean ± SD (n = 4). P values were calculated using one-way ANOVA followed by post hoc Bonferroni test. ns: no significance.

References

[1] X. Y. Dai, Y. Y. Zhang, L. N. Gao, T. Bai, W. Wang, Y. L. Cui, W. G. Liu, *Adv. Mater.* **2015**, 27, 3566.

[2] C. C. Fan, B. Liu, Z. Y. Xu, C. Y. Cui, T. L. Wu, Y. Yang, D. F. Zhang, M. Xiao, Z. D. Zhang, W. G. Liu, *Mater. Horiz.* **2020**, 7, 1160.

[3] F. Gao, Y. Y. Zhang, Y. M. Li, B. Xu, Z. Q. Cao, W. G. Liu, *ACS Appl. Mater. Interfaces* **2016**, 8, 8956.

[4] Q. Zhang, Z. Y. Xu, X. P. Zhang, C. J. Liu, R. Yang, Y. G. Sun, Y. H. Zhang, W. G. Liu, *Adv. Funct. Mater.* **2022**, 32, 2200360.

[5] P. Chansoria, J. Blackwell, E. L. Etter, E. E. Bonacquisti, N. Jasiewicz, T. Neal, S. A. Kamal, J. Hoque, S. Varghese, T. Egan, J. Nguyen, *Adv. Funct. Mater.* **2022**, 32, 2207590.

[6] C. Q. Zhao, P. C. Zhang, J. J. Zhou, S. H. Qi, Y. Yamauchi, R. R. Shi, R. C. Fang, Y. Ishida, S. T. Wang, A. P. Tomsia, M. J. Liu, L. Jiang, *Nature* **2020**, 580, 210.

[7] Y. Y. Zhang, Y. M. Li, W. G. Liu, *Adv. Funct. Mater.* **2015**, 25, 471.

[8] F. Gao, Z. Y. Xu, Q. F. Liang, B. Liu, H. F. Li, Y. H. Wu, Y. Y. Zhang, Z. F. Lin, M. M. Wu, C. S. Ruan, W. G. Liu, *Adv. Funct. Mater.* **2018**, 28, 1706644.

[9] Z. Y. Xu, Q. Zhang, C. C. Fan, M. Xiao, R. Yang, Y. Yao, Y. Wu, X. F. Nie, H. Y. Wang, W. G. Liu, *Bioact. Mater.* **2023**, 26, 64.

[10] A. D. Becke, *J. Chem. Phys.* **1993**, 98, 5648.

[11] C. Lee, W. Yang, R. G. Parr, *Phys. Rev. B* **1988**, 37, 785.

[12] F. Weigend, R. Ahlrichs, *Phys. Chem. Chem. Phys.* **2005**, 7, 3297.

[13] F. Weigend, *Phys. Chem. Chem. P*hys. **2006**, 8, 1057.

[14] S.Grimme, S.Ehrlich, L.Goerigk, *J. Comput. Chem*. **2011**, 32, 1456.

[15] A. V. Marenich, C. J. Cramer, D. G.Truhlar, *J. Phys. Chem. B* **2009**, 113, 6378.

[16] Z. D. Zhang, Q. N. Cao, Y. Xia, C. Y. Cun, Y. Qi, Q. Zhang, Y. H. Wu, J. F. Liu, W. G. Liu, *Bioact. Mater.* **2024**, 31, 408.

[17] K. P. Pritzker, S. Gay, S. A. Jimenez, K. Ostergaard, J. P. Pelletier, P. A. Revell, D. Salter, W. B. van den Berg, *Osteoarthr. Cartil.* **2006**, 14, 13.
